# Social and ecological complexity is associated with gestural repertoire size of wild chimpanzees

**DOI:** 10.1111/1749-4877.12423

**Published:** 2020-06-24

**Authors:** Sam G. B. ROBERTS, Anna I. ROBERTS

**Affiliations:** ^1^ School of Psychology Faculty of Health Liverpool John Moores University Liverpool UK; ^2^ Institute of Human Biology and Evolution Adam Mickiewicz University Poznań Poland; ^3^ Department of Psychology University of Chester Chester UK

**Keywords:** chimpanzee, ecology, gesture, repertoire size, sociality, social network analysis

## Abstract

Increasing our understanding of primate gestural communication can provide new insights into language evolution. A key question in primate communication is the association between the social relationships of primates and their repertoire of gestures. Such analyses can reveal how primates use their repertoire of gestural communication to maintain their networks of family and friends, much as humans use language to maintain their social networks. In this study we examined the association between the repertoire of gestures (overall, manual and bodily gestures, and gestures of different modalities) and social bonds (presence of reciprocated grooming), coordinated behaviors (travel, resting, co‐feeding), and the complexity of ecology (e.g. noise, illumination) and sociality (party size, audience), in wild East African chimpanzees (*Pan troglodytes schweinfurthii*). A larger repertoire size of manual, visual gestures was associated with the presence of a relationship based on reciprocated grooming and increases in social complexity. A smaller repertoire of manual tactile gestures occurred when the relationship was based on reciprocated grooming. A smaller repertoire of bodily gestures occurred between partners who jointly traveled for longer. Whereas gesture repertoire size was associated with social complexity, complex ecology also influenced repertoire size. The evolution of a large repertoire of manual, visual gestures may have been a key factor that enabled larger social groups to emerge during evolution. Thus, the evolution of the larger brains in hominins may have co‐occurred with an increase in the cognitive complexity underpinning gestural communication and this, in turn, may have enabled hominins to live in more complex social groups.

## INTRODUCTION

Understanding the evolution of language is one of the most important questions in establishing whether or not humans are truly distinct from other animals. In seeking to infer the evolution of language, a primary focus has been to understand communication in primates. As language is primarily vocal, most studies have focused on primate vocal communication. However, a theory of language evolution that is gaining increasing support is that language evolved not from vocalizations, but initially from gestures. Non‐human primates (hereafter primates), and particularly great apes, have an extensive repertoire of gestures, defined as voluntary movements of the arms, head, body postures and locomotory gaits (Liebal *et al*. 2004; Nishida *et al*. 2010; Liebal & Call 2012). While other species such as ravens (Pika & Bugnyar 2011), elephants (Moss *et al*. 2011) and black bears (Kilham 2013) use forms of gestural communication (Palagi *et al*. 2016), there has been an intensive research focus on great ape communication because of the importance of understanding primate communication in developing theories of human language evolution (Fitch 2010; Byrne *et al*. 2017; Corballis 2017). In particular, manual gestures, defined as movements of the hands without the use of objects or a substrate, have attracted considerable attention because of the possibility of being an ancestral trait that humans share with their primate relatives. It has been argued that manual gestures are governed by specific neurological structures homologous to the ones responsible for human language. Only humans and other apes habitually use their hands to communicate and gestural communication shows greater flexibility than either facial or vocal signals (Roberts *et al*. 2012b; Byrne *et al*. 2017). The homology between humans’ and apes’ manual gestures suggests that there was a relatively recent evolution of complex, manual gestures in human ancestors. One feature of this evolution is repertoire size, defined as the number of types of gestures made in a single utterance by the same individual towards the same recipient, within the same goal or context (“sequence repertoire size”). The repertoire size of communication signals is a key aspect to human communication and is part of what characterizes social relationships among humans as complex.

One central defining feature of complex social relationships in primates is grooming reciprocity. Unidirectional grooming (when one individual grooms another) builds closeness between partners which leads to grooming reciprocity. In socially bonded dyads, grooming reciprocity is underpinned by complex, coordinated interactions in many different contexts such as proximity, joint resting and travel. In primates, a larger repertoire of manual gestures can elicit accurate responses from the recipient, suggesting that a larger repertoire of manual gestures may enable primates to maintain more complex social relationships (Wittgenstein 1953; Roberts *et al*. 2014a). In contrast, a larger repertoire size of bodily gestures is related to reduced accuracy of responsiveness, perhaps reflecting similar neural processes underlying the use of communication as in humans (Roberts *et al*. 2014a). The outstanding question in the debate of the evolutionary antecedents of human social complexity is whether only humans use a large repertoire of manual gestures to maintain complex social relationships, or if the common ancestor of hominins and chimpanzees also had this ability. Evolutionarily speaking, together with the bonobos, chimpanzees are most closely related to humans, and, therefore, are a key species to study when searching for these abilities.

Studies of both captive and wild chimpanzees have demonstrated flexibility in gestural communication in relation to the recipient's response to the communication. For example, when there is a lack of desired response by the recipient to gestural communication, signalers elaborate on the initial gesture by using different gesture types, thus increasing the repertoire size of the gesture sequence (Leavens *et al*. 2005; Roberts *et al*. 2013). Where recipients are likely to be unresponsive, chimpanzees use gesture types that do not overlap with the repertoire of the recipient, suggesting that the nature of responsiveness is associated with repertoire diversity (Roberts *et al*. 2014a; Roberts & Roberts 2017).

Repertoire size is also associated with the nature of the relationship between the signaler and the recipient in chimpanzees (Roberts *et al*. 2019a). Chimpanzees are more likely to respond to a larger repertoire size that results in them being able to spend more time in proximity to others, and to obtain a central position in the social network (Roberts 2018; Roberts & Roberts 2018a). Furthermore, the dyadic repertoire size of visual and auditory short‐range gestures is associated with the duration of time that dyads spend in close proximity (within 10 m) (Roberts *et al*. 2019a). Chimpanzees with more proximity partners have a larger dyadic repertoire size and specifically a larger repertoire of visual gestures (Roberts *et al*. 2019a). While these results suggest that repertoire size is associated with overall sociality (measured by proximity), previous studies did not examine how repertoire size is associated with key social behaviors such as grooming and joint behaviors such as travel and feeding. More importantly, these previous studies did not differentiate between manual and bodily gestures, but such a distinction appears to be important as previous findings show differentiation in the repertoire size of manual and bodily gestures across social contexts (Roberts *et al*. 2014a).

The wider audience (i.e. individuals other than the signaler and the recipient who are present when gestural communication is occurring) may also influence repertoire size. The presence of an audience and the nature of that audience have a wide variety of effects on individuals’ behavior in both humans (Hamilton & Lind 2016) and in non‐human animals (Zajonc & Sales 1966; Mitani *et al*. 2002; Kaburu & Newton‐Fisher 2016). While audience effects have been studied in relation to grooming (Kaburu & Newton‐Fisher 2016) and vocalizations (Slocombe & Zuberbühler 2007) in primates, very little is known about how audiences affect gestural communication, apart from in a mating context (Roberts & Roberts 2015). A social audience composed of individuals that are the same‐age cohort as the interacting dyad provides other potential conspecifics that the dyad may want to interact and associate with (Kaburu & Newton‐Fisher 2016). This may draw the dyad partners’ attention away from each other and, thus, reduce the efficacy of communication between the signaler and the recipient (Mitani *et al*. 2002). For instance, when the audience of same‐aged chimpanzees as the recipient is larger, the lack of mutual interest in attention and proximity between the signaler and the recipient is associated with the use of left‐handed gestures (Isham & Geng 2011; Leary & Allen 2011; Roberts *et al*. 2019b). Thus, this form of sociality where large audiences are present may require an adjustment of repertoire size to enable complex social groups to emerge during evolution. However, how the repertoire size of manual and bodily gestures is shaped by the presence of the wider audience has not been examined.

Finally, some studies have proposed that ecology can influence repertoire size, as it can affect the ability of the signaler to influence the recipient. The effect of environmental factors on vocalizations in primates has been explored (Brown & Waser 1988; Mitani & Stuht 1998), but less is known about how these factors may affect gestural repertoires. Low levels of light, high levels of ambient noise and high levels of wind can all make it more difficult for the recipient to detect gestural communication from the signaler. A lower density of vegetation, while making detection of the gestural communication easier, may expose primates to a greater predation risk, potentially creating a greater need for vigilance that can influence the quality of communication. Furthermore, ambient temperature can affect activity patterns (Hill *et al*. 2004) and is also likely to affect rates of communication. In a study of mating gestures in chimpanzees, Roberts and Roberts (2015) found that when the wind intensity increased, the production of mating gestures decreased, providing some evidence that chimpanzees do adjust their gestural communication according to ecological factors for this specific behavioral context. These previous findings suggest that the complexity of the social environment and ecology may be associated with the repertoire size of gestures overall as well as manual and bodily gestures. Examining these associations may provide insights into whether flexibility in gestural communication may help chimpanzees meet the demands of living in a complex social group and in complex environmental conditions (Freeberg *et al*. 2012; Roberts & Roberts2016b).

In this study, we examine the associations between gestural repertoire size, sociality and ecological factors in wild East African chimpanzees (*Pan troglodytes schweinfurthii*) living in Budongo Forest, Uganda. Chimpanzees are an ideal species to examine these communication strategies because they live in complex, fission–fusion communities. In these societies, individuals maintain a differentiated set of social relationships of the type that is hypothesized to be associated with communicative complexity (Barrett *et al*. 2003; Amici *et al*. 2008; Freeberg *et al*. 2012). For instance, chimpanzees often encounter other group members at infrequent intervals and this may require adjustment of their communication strategies to the strength of the bond with the social partner. We first ask whether gestural communication towards recipients with whom the signaler has a relationship based on reciprocated grooming is associated with the complexity of the social and ecological environment. We predict that as social and ecological complexity increases, the greater processing demands on the recipient of grooming will imply that the grooming will be less likely reciprocated and, therefore, the signaler will be less likely to communicate towards partners who reciprocate grooming. We then ask the question: Do chimpanzees flexibly adjust communication strategies to account for the variation in the grooming reciprocity due to the increasing social and ecological complexity of their environment? To answer this question, we examine variation in repertoire size of gestures (overall, bodily and manual considered separately) within a single utterance (“sequence repertoire size”) in relation to social and ecological factors. We predict that as the social and ecological complexity increases, chimpanzees will adjust the repertoire size of gestures to facilitate social bonding, based on reciprocated grooming in more complex social and ecological settings.

The second key question addressed in this study is which characteristics of sociality and communication drive “individual repertoire size” (the total number of gesture types in a chimpanzee's repertoire that is directed at other adult conspecifics). Homophily (attraction to individuals who possess similar characteristic to oneself such as personality or age) influences sociality in primates (Massen & Koski 2014). When small audiences are present, chimpanzees may interact with all group members and, thus, the influence of homophily may be less apparent. In contrast, when large audiences are present, chimpanzees may interact with conspecifics who possess a similar repertoire size (McPherson *et al*. 2001). If chimpanzees interact in complex social settings with individuals who possess a similar repertoire size, then we predict that the communication between dyad partners will have characteristics of the type of communication seen in complex social settings, such as tactile and auditory gestural communication, rather than visual gestures. Moreover, we predict that if homophily for repertoire size guides social behavior in primates, then we should observe associations between individual repertoire size and social behaviors known to characterize close social bonding (e.g. mutual grooming, joint travel and mutual visual monitoring).

The final research question we address in this paper is whether the number of bonded social partners that individuals have in their network is related to the repertoire size produced by those individuals, or received by those individuals (“dyadic repertoire size”). If a larger repertoire size of gestures enables chimpanzees to be more successful in establishing and managing social relationships with central individuals in the network than a smaller repertoire size, then we would expect that the individuals who hold a central position in the network will receive a larger repertoire size of gestures.

## MATERIALS AND METHODS

### Study site and subjects

Observations were made on the Sonso community (6 adult males and 6 adult females) of East African chimpanzees (*Pan troglodytes schweinfurthii*) at the Budongo Conservation Field Station, Bundongo Forest Reserve in Uganda, East Africa. On average we observed each focal subject for 12 h, with focal observation ranging between 8.33 h and 18.63 h. The duration of observation of each focal subject is given in Table S1. Focal chimpanzees did not display any limb injuries and they were well habituated for detailed behavioral observation. Full details of the study site, subjects, data collection, video analysis and classification of the gestures have been described previously (Roberts *et al*. 2012a,b, 2013, 2014a), so only brief details are given here. The research was non‐invasive and was approved a priori by the University of Stirling Ethics Committee; it followed the Association for the Study of Animal Behaviour ethical guidelines.

### Data collection

Using focal animal follows, we recorded social relationships and communication during randomly selected sessions of focal observation. We recorded the behavior of the focal and non‐focal individuals who were present in the same party. The term “party” was operationalized as a group of individuals within a spread of 35 m. First, 9 scans, each at 2‐min intervals (total duration 18 min) recorded individuals that were present within 10 m of the focal subject and also individuals more than 10 m away that were present in the same party, the identity of the adult nearest neighbor, their proximity in meters and bodily orientation relative to the focal subject. The activity of the focal subject and the nearest neighbor were recorded. Second, 3 scans, each at 6‐min intervals (total duration 18 min) recorded ecology alongside the social scans. We recorded noise, illumination, wind, visibility, visitor number and visitor distance. On the third scan we also recorded temperature. The methods of recording illumination, ambient noise, temperature and wind are outlined in Roberts and Roberts (2015). Visitor distance was recorded as the distance between the focal individual and the human who was closest to the focal individual. If the focal subject was above the ground, the hypotenuse (not height) was recorded to indicate distance. Visitor number was recorded as an overall number of all visitors present within a 30‐m diameter of the chimpanzees. For visibility, the greatest horizontal distance at which waving of an arm of the chimpanzee would be visible to the focal animal was recorded in 4 cardinal directions and mean of 4 directions was calculated. Finally, we continuously recorded gestures using a digital video camera. When gestures were observed, the behavior of the signaler and recipient along with the context of the signal production were recorded. The detailed method for deriving the inventory of gesture types from the video recordings were described previously (Roberts *et al*. 2012a,b, 2013, 2014a; Roberts & Roberts 2015). Validation of the coding procedure was established by a second coder who scored a random sample of 45 of the sequences of gestures for concordance in function and modality. The Cohen's kappa coefficient showed that reliability was good for function (*K* = 0.70) and modality of gesturing (*K* = 0.946) (Bakeman & Gottman 1997). A different sample of 50 sequences of gestures was coded by a second coder for intentionality (response waiting and persistence) and the Cohen's kappa coefficient showed good reliability (*K* = 0.74). The sampling of behavior was done by an experienced field assistant who was unaware of the aims of the study. An inter‐observer reliability test of the chimpanzee identities and proximities is conducted annually to maintain the consistency of the scoring of the group composition and proximity across field assistants. The Spearman's rank correlation coefficient for this test is at least or above 0.85.

### Behavioral data

In this work we only took into account those instances of gestural communication when the intended recipient of the gestural communication was within 10 m of the signaler. The distance of 10 m was chosen to take into account the ability of the recipient to perceive the signal in a dense forest habitat (Roberts & Roberts 2016b). To ensure the independence of the sampling procedure, we examined similarity in association patters within and between the samples: this was described in detail in Roberts and Roberts (2016a). These analyses showed that the sampling procedure was independent between the samples but dependent within the sample. The behavioral indices derived from these scans (e.g. duration of proximity) were described in Roberts and Roberts (2016a,b). Similarly, the attribute measures (age, kinship, sex and reproductive state) were previously described (Roberts & Roberts 2016a,b).

### Social network analysis

Definitions and descriptive statistics for variables entered into social network analyses can be found in Table S2. We created social and communication weighted networks. Each network had 12 rows and 12 columns (representing the 12 focal chimpanzees) and the value in each cell of the matrix denotes the value for a particular behavior exhibited by the specific pairs of chimpanzees. Thus, the 12 × 12 matrix consisted of 132 dyads (144 minus 12). For the repertoire of gestures, the values in each cell relate to the gestural repertoire size produced by the focal chimpanzee and directed at a specific individual, per hour spent within 10 m of that individual. In these directed social networks, outdegree refers to behaviors directed by the focal chimpanzee to conspecifics, while indegree refers to behaviors directed by conspecifics towards the focal chimpanzee. For data transformation and analysis, we used UCINET 6 for Windows (Borgatti *et al*. 2014). For the node level regressions, we used 10 000 random permutations to assess the effect of several predictor variables (such as the outdegree of gestures and sex of focal chimpanzee) on the outcome variable (e.g. the mutual grooming outdegree). Finally, we used Geary's *C* statistics to examine the autocorrelation between attribute data (the total number of gestures in a focal chimpanzees’ repertoire) and network data (e.g. duration of proximity). This statistic has a value of 1 for no association, with values of less than 1 indicating a positive association and values of more than 1 indicating a negative association. Geary's *C* statistic was also used to examine the autocorrelations between the total duration of observation for each focal chimpanzee and repertoire network. There was not a statistically significant relationship between the total duration of observation and the repertoire network (*C* = 1.054, *P* = 0.397), suggesting sufficient sampling duration.

### Generalized linear mixed models

We used generalized linear mixed models (GLMM) to examine the factors influencing repertoire size recorded for each communication event. For each gesture entry, the attribute data (e.g. sex, age, kinship and estrous) was entered, social characteristics between dyad (i.e. presence or absence of grooming reciprocity, duration of joint travel, resting and feeding when dyad partners are nearest neighbours and within 2 meters of each other per hour spent in the same party), audience (i.e. number of same‐age partners as the signaler in the audience, number of same‐age partners as the recipient in the audience and total audience size), ecology (i.e. noise, illumination, temperature, wind, visibility, visitor number, visitor distance, proximity to signaler and recipient bodily orientation). The descriptive statistics regarding variables included in the GLMM are provided in Table S3. The mean Variance Inflation Factor (VIF) for continuous variables was 1.36, ranging between 1.10 and 1.76. When examining predictors of repertoire size based on social and ecological characteristics, the repertoire size of manual auditory short‐range gestures and the repertoire size of bodily tactile gestures were not considered separately due to small sample size. In the GLMM, the data was hierarchically structured with 2 levels: Level 1 was the focal individual and Level 2 was the recipient of the gesture. These models represent a form of a regression where the data has a hierarchical clustering structure. The model was fitted using a binomial error structure with logit link. The random effects included were the focal individual identity and the focal individual identity by recipient identity: for these effects, random intercepts were used. All analyses were carried out using IBM SPSS Statistics 22. Only significant associations are reported in the results section. Details of all models can be found in Tables S4–S29.

## RESULTS

### Is the duration of visual attention and proximity associated with the size and nature of the audience?

We used GLMM to examine predictors of audience size (i.e. number of same‐age partners of the signaler in the party, number of same‐age partners of the recipient in the party, total number of chimpanzees in the party: Tables S4–S9) based on the duration of time spent in proximity to 2 m and mutual visual monitoring. Chimpanzee dyads who gestured in the presence of a larger number of same‐age partners as the recipient spent a shorter duration of time in proximity to 2 m (β = −0.010, *P* < 0.001) and in mutual visual monitoring (β = −0.014, *P* < 0.001).

### Is the likelihood of response to the gesture (present or absent) associated with the nature of social relationship and sequence repertoire size?

When dyads did not reciprocate grooming, chimpanzees were more likely to change behavior in response to a larger repertoire of manual gestures in the sequence (β = 1.466, *P* = 0.025), a larger repertoire size of manual auditory long‐range gestures (β = 1.724, *P* = 0.019) and a larger repertoire size of manual auditory short‐range gestures (β = 1.322, *P* < 0.001) but a smaller repertoire size of bodily auditory short‐range gestures (β = −2.097, *P* < 0.001). When dyads reciprocated grooming, a response was more likely when the signaler produced a larger repertoire size of bodily tactile gestures (β = 11.505, *P* < 0.001), a smaller repertoire size of bodily auditory long‐range gestures (β = −1.248, *P* < 0.001) and a smaller repertoire size of bodily auditory short‐range gestures (β = −5.463, *P* = 0.001). See Tables S10–S15 for details of these findings.

### Is communication between dyads who reciprocated grooming associated with social and ecological conditions?

Table S16 shows details of this model. Dyads who reciprocated grooming gestured more frequently towards partners of the same age (β = −17.246, *P* < 0.001), different sex (β = 22.115, *P* = 0.002), maternal kin (β = −131.427, *P* = 0.005) and reproductively active (β = −15.536, *P* < 0.001) recipients, with whom they spent a longer duration of time in joint feeding (β = 0.962, *P* = 0.042) and travel (β = 3.042, *P* = 0.004). Dyads who reciprocated grooming were more likely to gesture when the illumination was higher (β = 0.001, *P* = 0.027), the noise levels were lower (β = −0.404, *P* = 0.038), the visibility was lower (β = −0.308, *P* = 0.045), visitor numbers were lower (β = −4.886, *P* < 0.001), the audience of same‐age partners as the focal individual was larger (β = 7.242, *P* < 0.001), the audience of same‐age partners as the recipient was smaller (β = −5.849, *P* = 0.002) and total party size was smaller (β = −1.731, *P* < 0.001). Dyads who reciprocated grooming gestured when the recipient was closer (β = −0.998, *P* = 0.004), using a larger repertoire of bodily auditory long‐range gestures (β = 8.022, *P* = 0.016), a larger repertoire of manual visual gestures (β = 5.394, *P* = 0.003), a smaller repertoire of bodily auditory short‐range gestures (β = −6.198, *P* = 0.041), a smaller repertoire of manual auditory long‐range gestures (β = −3.986, *P* = 0.033) and a smaller repertoire of manual tactile gestures (β = −5.797, *P* = 0.014).

### Is sequence repertoire size associated with social and ecological factors?

**Figure 1:**
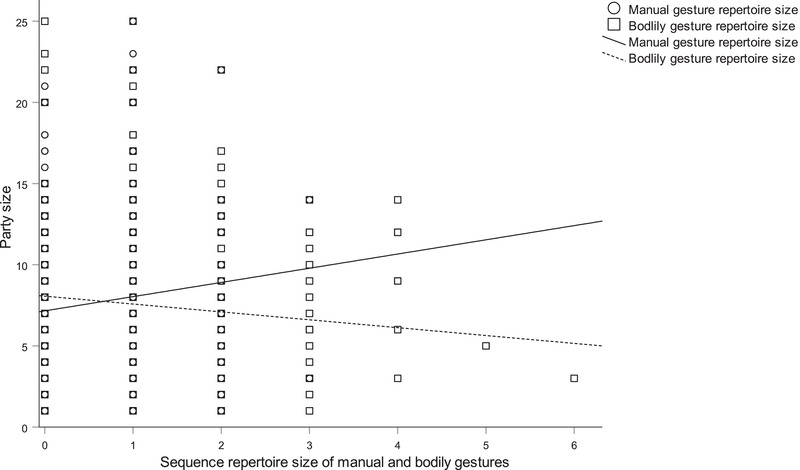
Scatterplot (with lines of best fit) of the relationship between party size, sequence repertoire size of manual gestures (open circles and solid line) and sequence repertoire size of bodily gestures (open squares and dotted line).

**Figure 2:**
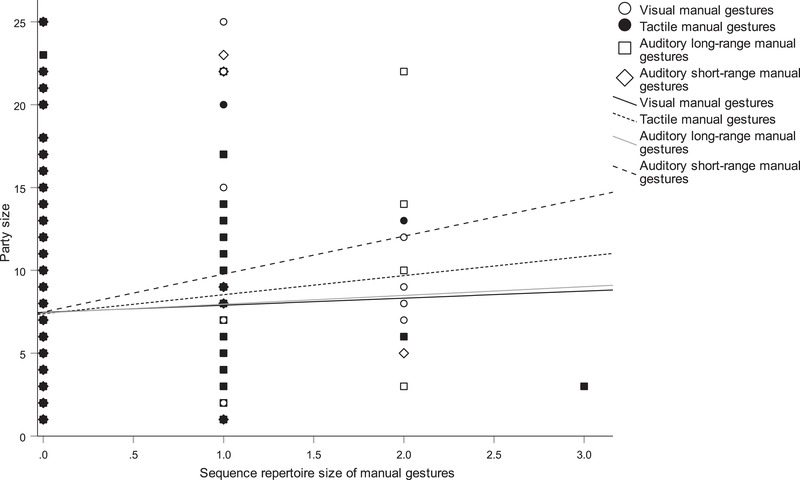
Scatterplot (with lines of best fit) of the relationship between party size and sequence repertoire size of manual gestures by modality: visual gestures (open circles and solid line), tactile gestures (solid circle and dashed line), auditory long‐range gestures (open square and grey line) and auditory short‐range gestures (open diamond and dashed line).

In Analysis 1, social and ecological predictors of the overall repertoire size of gestures were examined (Table S17). A larger repertoire size was associated with gesturing towards non‐kin (β = 1.289, *P* = 0.022) and reproductively inactive partners (β = 1.146, *P* = 0.031), when distance to the recipient was larger (β = 0.108, *P* < 0.001), when temperature was lower (β = −0.061, *P* < 0.001), when duration of joint travel was shorter (β = −0.119, *P* = 0.009), and when the audience size of same‐age partners as the focal subject was larger (β = 0.342, *P* = 0.028). Analysis 2 examined the associations between manual repertoire size and social and ecological factors (Table S18). A larger repertoire size of manual gestures was associated with gesturing towards different‐age partners (β = 0.375, *P* = 0.007), non‐kin (β = 0.692, *P* < 0.001) and reproductively inactive partners (β = 0.621, *P* = 0.005). A larger repertoire size of manual gestures was also associated with a larger distance to the recipient (β = 0.042, *P* < 0.001), a larger number of visitors (β = 0.075, *P* < 0.001), a greater distance to the visitors (β = 0.009, *P* = 0.023), a longer duration of joint resting (β = 0.091, *P* = 0.006) and when the audience size of same‐age partners as the focal subject was larger (β = 0.355, *P* = 0.001). Analysis 3 examined the predictors of the repertoire size of bodily gestures (Table S19). Chimpanzees directed a larger repertoire of bodily gestures at the partner when the distance to the recipient was larger (β = 0.059, *P* = 0.001), the temperature was lower (β = −0.052, *P* < 0.001) and the duration of joint travel was shorter (β = −0.111, *P* < 0.001; Fig. 1). In Analysis 4, looking at predictors of manual visual repertoire size (Table S20), we found that chimpanzees used a larger repertoire of these gestures when the recipient was of a different age (β = 0.182, *P* = 0.002) or same sex (β = −0.408, *P* < 0.001), the illumination was lower (β = −0.001, *P* < 0.001), the number of visitors was higher (β = 0.066, *P* < 0.001), the distance to visitors was higher (β = 0.015, *P* < 0.001), the number of same‐age partners as the recipient in the audience was smaller (β = −0.172, *P* = 0.005), the number of same‐age partners as the signaler in the audience was larger (β = 0.209, *P* = 0.030), the total audience size was larger (β = 0.014, *P* = 0.019) and the recipient of gesturing reciprocated the signaler's grooming (β = −0.263, *P* < 0.001). In Analysis 5, the sequence repertoire size of manual, tactile gestures (Table S21) was significantly positively associated with different‐age partners (β = −0.245, *P* = 0.001), reproductively inactive partners (β = 0.424, *P* = 0.040), lower noise (β = −0.003, *P* = 0.015), a longer duration of joint resting (β = 0.072, *P* < 0.001), a longer duration of joint travel (β = 0.036, *P* < 0.001), larger audience size of same‐age partners as the recipient (β = 0.113, *P* = 0.012) and lack of reciprocity in grooming (β = 0.197, *P* = 0.017). Analysis 6 (Table S22) showed that when the sequence repertoire size of manual, auditory long‐range gestures increased, chimpanzees directed gestures at different sex partners (β = 0.429, *P* = 0.009), the distance to recipient was longer (β = 0.026, *P* = 0.041), the temperature was lower (β = −0.026, *P* = 0.004), the visibility was higher (β = 0.018, *P* < 0.001), the distance to visitors was smaller (β = −0.014, *P* < 0.001), the duration of travel was shorter (β = −0.045, *P* = 0.047) and the party size was smaller (β = −0.018, *P* = 0.021; Fig. 2).

**Figure 3:**
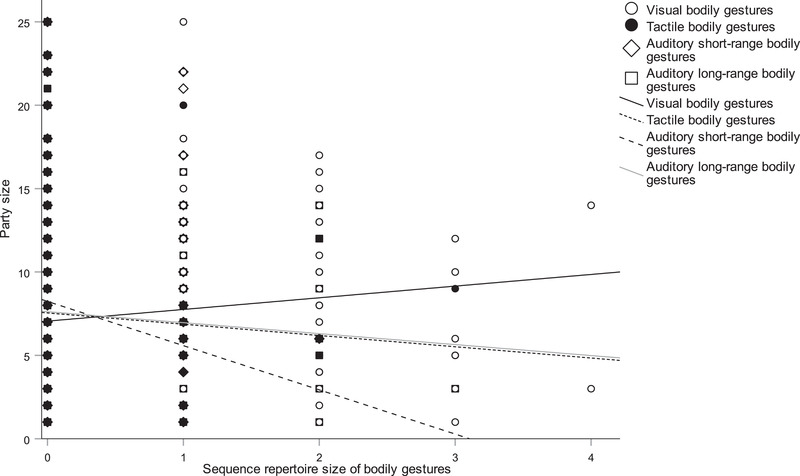
Scatterplot (with lines of best fit) of the relationship between party size and sequence repertoire size of bodily gestures by modality: visual gestures (open circles and solid line), tactile gestures (solid circle and dashed line), auditory short‐range gestures (open diamond and dashed line) and auditory long‐range gestures (open square and grey line).

Analyses 7, 8 and 9 examined which social and ecological variables predicted the repertoire size of bodily gestures in the visual, auditory long‐range and auditory short‐range modalities. (Tables S23–25). Analysis 7 showed that a larger distance to recipient (β = 0.067, *P* < 0.001), lower temperature (β = −0.044, *P* < 0.001) and shorter duration of joint travel (β = −0.061, *P* = 0.001) predicted a larger repertoire of bodily visual gestures. According to Analysis 8, chimpanzees who used a larger repertoire of bodily auditory long‐range gestures communicated when visibility was higher (β = 0.011, *P* = 0.009) and when the duration of joint travel between partners was shorter (β = −0.038, *P* = 0.009). Finally, in Analysis 9 there was a positive association between the repertoire size of bodily auditory short‐range gestures and use towards same‐age partner as the recipient (β = −0.539, *P* < 0.001), shorter distance towards the recipient (β = −0.030, *P* < 0.001), lower wind (β = −0.341, *P* = 0.008), smaller visitor distance (β = −0.022, *P* = 0.021), shorter duration of joint resting (β = −0.063, *P* < 0.001) and travel (β = −0.054, *P* < 0.001), and the recipient facing the signaler with their back (β = 0.097, *P* = 0.031; Fig. 3).

### Dyadic repertoire size and sociality centrality

The next set of analyses used node‐level regressions to examine the predictors of sociality centrality by repertoire size of gestures (Tables S26–S27). In these analyses we controlled for the duration of time spent in proximity to estrus females, time spent in proximity to kin, and the age and sex of the focal chimpanzee. The analyses showed that the chimpanzees with a high indegree of proximity (β = 0.672, *P* = 0.047), travel (β = 0.738, *P* = 0.031), give grooming (β = 0.653, *P* = 0.047), mutual grooming (β = 0.794, *P* = 0.026) and attention toward (β = 0.667, *P* = 0.048), had a high indegree of repertoire size. In contrast, individuals who had high outdegree of proximity (β = 0.764, *P* = 0.047), rest (β = 0.756, *P* = 0.047) and attention away (β = 0.791, *P* = 0.037) had a high outdegree of repertoire size.

### Individual repertoire size and sociality

We used Geary's *C* statistic to examine the autocorrelations between the individual repertoire size of gestures of each focal chimpanzee, the demography and the duration of time spent in the social activities network (Table S28). First, chimpanzees who tended to have a similar repertoire size were the same age (*C* = 0.408, *P* = 0.020) and same sex (*C* = 0.167, *P* = 0.002), non‐kin (*C* = 2.208, *P* = 0.021) and reproductively inactive dyads (*C* = 1.472, *P* = 0.036). Second, the analyses revealed that the chimpanzees who tended to have a similar repertoire size of gestures to each other spent a longer duration of time engaged in travel (*C* = 0.425, *P* = 0.035), and had greater attention present (*C* = 0.499, *P* = 0.006), grooming given (*C* = 0.474, *P* = 0.036), grooming mutual (*C* = 0.382, *P* = 0.048) and grooming received (*C* = 0.400, *P* = 0.022).

### Individual repertoire and communicative complexity

Finally, we used Geary's *C* statistic to examine the autocorrelations between the individual repertoire size of gestures of each focal chimpanzee and the rate of production of gestures classified according to complexity (Table S29). The analyses revealed that the chimpanzees who tended to have a similar repertoire size to each other directed a higher rate of following gesture types at each other: bodily (*C* = 0.498, *P* = 0.011), non‐combined (*C* = 0.500, *P* = 0.017), events (*C* = 0.596, *P* = 0.034), no‐object (*C* = 0.461, *P* = 0.006), multimodal with high amplitude vocalization (*C* = 0.436, *P* = 0.047), auditory short‐range (*C* = 0.188, *P* = 0.009), tactile (*C* = 0.197, *P* = 0.003), dyadic repertoire size (*C* = 0.601, *P* = 0.041), multimodal with facial expression (*C* = 0.236, *P* = 0.022), multimodal with vocalization (*C* = 0.541, *P* = 0.038), unimodal (*C* = 0.512, *P* = 0.025), mutual attention present (*C* = 0.403, *P* = 0.004), mutual attention absent (*C* = 0.320, *P* = 0.007), close proximity (*C* = 0.221, *P* = 0.001), homogenous (*C* = 0.350, *P* = 0.001), non‐repetitive (*C* = 0.584, *P* = 0.040), repetitive (*C* = 0.504, *P* = 0.015), single (*C* = 0.420, *P* = 0.004), response present (*C* = 0.433, *P* = 0.002), response absent (*C* = 0.298, *P* = 0.001) and response by activity change (*C* = 0.480, *P* = 0.004).

## DISCUSSION

Previous studies of gestural communication of primates have identified different patterns of response specificity to gestures overall, and across manual and bodily gestures. Here we extend these findings to show that the repertoire size of manual and bodily gestures of wild chimpanzees is differentiated in the nature of its association with social relationships and ecology.

First, we asked the question: Is the likelihood of communication towards partners who reciprocated grooming influenced by the complexity of the social and ecological environment within which the dyad interacts? We found that communication towards partners who reciprocated grooming was associated with social and ecological complexity. When the party size increased and when the number of same‐age partners as the recipient in the party increased, chimpanzees were less likely to communicate with the partners who reciprocated grooming. Furthermore, when the distance to the recipient increased, when noise increased, when illumination declined, when visitor number increased and when visibility increased, chimpanzees were less likely to communicate with the partners who reciprocated grooming. With increasing social and ecological complexity, there is pressure on the recipient's processing abilities to infer meaning from the behavior and this may influence the reciprocity of grooming. In particular, it may be more difficult for recipients to distinguish between potential goal states of the signaler when the complexity of the immediate audience increases (Zajonc & Sales 1966). Thus, as the cognitive demands of processing information increase, the speed and accuracy of responses by the recipient may decline, resulting in less efficient social bonding (Chittka *et al*. 2009). This finding suggests that social and ecological complexity creates pressure on the signaler to evolve cognitively complex communication strategies underpinned by an awareness of the strength of the social bond with the partner and the ability to respond adaptively by adjusting communication strategies to the complexity of the social and ecological setting (Dunbar 2012; Bohn *et al*. 2019).

Given the complexity of cognitive abilities of the chimpanzees in the social domain, it is important to examine whether different communication strategies differ in their effectiveness of influencing social bonding, in order to provide an empirically grounded account of the evolutionary predecessors to increases in the complexity of social structure of hominins, alongside increases in cognitive and communicative complexity. Our study shows that chimpanzees adjusted their repertoire of bodily gestures in the context of travel. When chimpanzee dyads traveled together for longer, this co‐occurred with a smaller repertoire of bodily gestures directed at the partner. However, in contrast with manual gestures, the smaller repertoire size of bodily gestures may have been associated with the presence of unreciprocated bonds. This finding supports the view that manual gestures may be particularly important in the development of complex social relationships based on reciprocated grooming (Pollick & de Waal 2007). By reducing the repertoire size of bodily gestures within the sequence, signalers may increase the specificity of the response, indicating that chimpanzees have voluntary control over the use of bodily gestures (Roberts *et al*. 2014a). The association between repertoire size of manual gestures and measures of social complexity may indicate the ability of the recipient to flexibly associate signals with the referents in response to pressures created by the complexity of the social environment, such as audience size, and, hence, an ability to flexibly influence the outcome of social interactions. For instance, chimpanzees may associate the signal with a specific place on the body towards which to act (e.g. climb on the back rather than the chest) (Kita 2009). Ontogenetically acquired signal‐referent links can increase the specificity of the response by the recipient to emotional displays, as opposed to emotional signals that do not contain specific information (Chittka *et al*. 2009). Emotional displays such as bodily movements, left‐handed movements, facial expressions or vocalizations frequently co‐occur with intentional manual gestures. Because a large repertoire of manual gestures was found to be more specific to the response by the recipient than a large repertoire of bodily gestures, this suggests that manual gestures may play a role in influencing the ability of the recipient to attribute referent to emotional bodily gestures and hence social bonding between the signaler and the recipient (Roberts *et al*. 2012b, 2014a).

The ability to flexibly associate emotional display with the referents during instances of social interactions with conspecifics may play a key role in facilitating social complexity through increasing specificity of the response by the recipient (Roberts *et al*. 2019b). Ontogenetically encoded emotional signals may be important in social coordination when social complexity increases because they might not demand contextual evaluation of the signal's meaning, but may elicit largely automatic but specific reactions to the emotional behavior. It is hypothesized that when primates interact in a simple social environment, recipients can make associations between the emotional display and the referent individually from the ongoing context. When social complexity increases, however, the presence of a wider audience may cause disruption to the learning of the emotional display‐referent links. It was recently proposed that signalers evolved a number of strategies to increase the efficiency of learning of these links by drawing attention to the referent and increasing the ability of the recipient to make associations between emotional display and the referents. These strategies include, for instance, communicative persistence through right handed manual gestures and rewarding communication (e.g. mutual attention). These behaviors may reduce the cognitive demands on contextual comprehension of emotional display when social complexity increases, by making referents more apparent to the recipient and, hence, facilitating learning (Roberts & Roberts 2020). Here we further hypothesize that an adjustment of gestural repertoire size might influence the ability of the recipient to detect and respond to the communication. A larger repertoire of manual visual gestures was associated with the presence of a reciprocated relationship and greater social complexity. This contrasts with a smaller repertoire size of manual tactile gestures seen in association with reciprocated relationships. A larger repertoire of manual, visual gestures may enable chimpanzees to increase specificity within an utterance and, thus, increase the likelihood that the recipient will appropriately respond to the gesture. In contrast, tactile gestures may be more effective than visual gestures in directing the movement and attention of the recipient to the referent and, thus, only smaller repertoires may be required. Whereas repetition of the signals within the sequence reflects the internal emotional state of the signaler, elaboration of signals within the sequence identifies the signaler's awareness of the impact of communication on the recipient and, hence, capacity for cognitively complex, intentional communication (Bates *et al*. 1979; Tomasello *et al*. 1985; Golinkoff 1986, 1993).

In trying to understand the origins of the complex social structure of humans, a central question is whether the cognitive skills underlying communication in primates play a role in social complexity. This issue is important because the formation and maintenance of social bonds is cognitively demanding and plays a key role in the cohesion of primate society (Dunbar 2010). For instance, chimpanzees who communicated with partners who reciprocated grooming engaged in social coordination in contexts such as joint feeding and travel for longer than the partners who did not reciprocate grooming. Here we provide the first empirical evidence that the complex repertoire of manual visual gestures can underpin the emergence of complex social structure in wild chimpanzees. Studies show that the transition to complex sociality of hominins was first preceded by the evolution of pair‐bonded social system in primates, and then bonded social relationships with unrelated conspecifics outside of mating contexts within large and complex social groups (Shultz *et al*. 2011). However, it is unclear which factors have facilitated the transition to these complex forms of social living in our hominin ancestors. Here we suggest that this complex social structure may have been facilitated by more complex use of manual, visual gestures. These findings have parallels with the use of manual gestures in humans, where manual gestures are used flexibly to improve the efficiency of communication with the recipient. For example, children who are deaf use a variety of gestural communication when playing and adjust their gestures according to whether they are playing with deaf or hearing children (Lederberg *et al*. 1986). In adults, experimental work has demonstrated that when working in larger groups on a foraging task, use of gestures increased (Oesch & Dunbar 2018), while pointing appears to be specifically tuned to the recipient, taking their perspective in mind (Winner *et al*. 2019). The flexible use of manual gestures thus appears to lead to increased efficiency of communication in humans, supporting theories proposing a role of gestural communication in the evolution of human language (Corballis 2018).

While chimpanzees appear to effectively use gestural communication to facilitate social bonding in a complex social environment, communicative interactions also take place in variable conditions of ecological complexity. Our study shows that ecological variables were differently associated with various measures of repertoire complexity. For instance, a larger repertoire of manual visual gestures occurred when illumination declined and when there was a larger number of visitors observing a party. In contrast, a smaller repertoire of auditory long‐range bodily gestures occurred when the temperature increased, but a larger repertoire occurred when visibility increased. This indicates that different ecological conditions may impose different constraints on the processing abilities of the interacting dyad. It suggests that some of the ecological factors put greater pressure on the ability of chimpanzees to cope with environmental challenges than others. For instance, when there is a high temperature, the metabolic costs of processing information increase, and this appears to be associated with a decreased repertoire size (Kosheleff & Anderson 2009). In habitats with a high temperature, chimpanzees may respond to higher metabolic costs of processing information by decreasing gestural diversity. The cultural diversity of humans has been shown to be shaped by social and ecological characteristics (e.g. environmental disturbance, high temperature and population density) (Collard & Foley 2002). By examining how these factors affect gestural communication across primate species, future studies will illuminate the evolutionary antecedents to linguistic and cultural diversity.

Another key question addressed in this study was whether chimpanzees have a homophily for “individual repertoire size” (total number of gesture types that one has in their repertoire that are directed at adult conspecifics). There was a link between individual repertoire size and social interactions. Individuals who had a similar repertoire size engaged in social bonding behavior such as mutual grooming, joint travel and mutual visual monitoring for longer, as compared to individuals that had a dissimilar repertoire size. Thus, chimpanzees differentiated their social behaviour towards partners depending on similarity in repertoire size. This would suggest that individual repertoire size in wild chimpanzees might be important marker of strenght of social bonding within dyad. A link between “centrality repertoire size” and sociality further suggests that sociality may be interdependent with gestural repertoire size.

Chimpanzees that communicated with partners who had a similar repertoire size used tactile and auditory short‐range gestures, but also used multimodal signals such as gestures accompanied by facial expressions and high‐amplitude calls.

In large social groups when large audiences of “friends” such as same‐age partners as the recipient are present, humans choose among a large number of partners to interact with preferred conspecifics (McPherson *et al*. 2001). However, in large groups there may be time and cognitive constraints on ability to socially bond with conspecifics through one on one interactions leading to diverse strategies for social bonding.

Like in humans, one strategy that the chimpanzees may use in complex social settings is increasing the complexity and diversity of component parts of the gestures (Hopkins & Cantero 2003; Leavens *et al*. 2010; Genty *et al*. 2014). Both wild and captive chimpanzees use multimodal signal combinations to influence recipients (Crockford & Boesch 2003; Leavens *et al*. 2010; Genty *et al*. 2014). Whereas manual gestures can influence social bonding in dyadic one‐on‐one interactions, the rewarding and high intensity multimodal, bodily communication may facilitate social interactions on a larger scale, especially during travel or when approaching feeding sites (Tarr *et al*. 2014; Roberts & Roberts 2018b).

Future studies need to account for demographic differences in these analyses to exclude the possibility that these associations between individual repertoire size and social and communicative complexity are driven by demography. For instance, it may be reasonable to assume that males preferentially associate with each other, and have a larger repertoire size than females (Roberts & Roberts 2019) and, thus, the perceived homophily for repertoire size is driven by the preference for affiliation with same‐sex partners. Examining the factors influencing individual repertoire size may be informative regarding the aspects of chimpanzee communication most valuable for learning gesture repertoire. As well as looking at demography, future studies may test whether certain features of manual and bodily gestures considered separately best predict individual repertoire size.

A key characteristic of language is the ability to combine a large repertoire of signals within an utterance to create a large, open‐ended set of meanings that increase the ability of the recipient to respond adaptively (Bickerton 1987; Pinker & Bloom 1990; Fitch *et al*. 2005). Language is hypothesized to have evolved to enable humans to maintain more complex social relationships within large and dynamic social groups (Aiello & Dunbar 1993). Examining how the repertoire of gestures is related to sociality in chimpanzees, our closest living relatives, may enable us to gain insights into the evolution of language (Corballis 2003; Arbib *et al*. 2008). Our results are the first empirical demonstration of the association between the large repertoire of manual, visual gestures and social complexity in chimpanzees, suggesting that gestural communication may have played an important role in language evolution. This finding is supported by other research which shows the importance of manual, visual gestures in regulating social dynamics in primates (Pollick & de Waal 2007; Bohn *et al*. 2016; Roberts & Roberts 2018b; Roberts *et al*. 2019b) and humans (Lederberg *et al*. 1986; Liebal *et al*. 2009; Oesch & Dunbar 2018; Winner *et al*. 2019). More broadly, the gestural repertoire size of primates varies widely among species, ranging, for instance, from just 29 gesture types in the orangutans (Liebal *et al*. 2006) to at least 100 gesture types in the chimpanzees (Liebal *et al*. 2004; Nishida *et al*. 2010; Roberts *et al*. 2012b, 2014a; Pollick & de Waal 2007). A key debate in relation to the evolution of gestural communication is how this variation in repertoire size evolves (Dawkins & Guilford 1997). Some hypotheses suggest that gestural repertoires are under genetic control, and phylogenetic relationships determine the make‐up of the gesture repertoire both within and across species (Byrne *et al*. 2017). Other hypotheses suggest that repertoire size is shaped by the needs of social bonding in complex social and ecological conditions (Dawkins & Guilford 1997; Roberts & Roberts 2015; Roberts *et al*. 2019a). However, if gesture repertoires are genetically fixed, it is unclear why there is variation in repertoire size in relation to social and ecological conditions. Our study suggests that the similarity of challenges imposed by complex sociality and ecology may explain the similarity in gestural repertoire across great ape species. Future studies across different primate groups and species need to explore one key explanation for repertoire diversity: that the complexity of sociality and ecology shapes gestural repertoire size.

To summarize, the results suggest that the variation in repertoire size in the chimpanzee group can be accommodated within a parsimonious explanation of social bonding, driven by challenges imposed by complex sociality and ecology. Supporting previous research on the link between repertoire size and sociality in animals (Roberts & Roberts 2017; Konrad *et al*. 2018) and humans (Lederberg *et al*. 1986; Ginsborg & King 2009; Oesch & Dunbar 2018), we found that social context and ecology explained much of the variation in repertoire size. Chimpanzees who used a larger repertoire of manual visual gestures were more likely to engage in reciprocated interactions in complex social settings. In contrast, chimpanzees who used a smaller repertoire of bodily gestures coordinated their social activities during travel.

The social brain hypothesis suggests that the complex social world of primates is particularly cognitively demanding and this imposed an intense selective pressure for the evolution of increasingly larger brains (Dunbar & Shultz 2017). The size of the group in primates is strongly correlated with the size of the brain, but what makes the social environment of some species more “complex” than others is still poorly understood. The results presented here suggest that challenges of social bonding in complex social and ecological settings may be one factor that causes some social environments of primates to be more complex than others (Roberts 2018). These findings clearly indicate that the chimpanzee gesture repertoire is flexibly employed to manage the processing demands of social interactions in complex social and ecological settings.

## Supporting information


**Table S1** Identity of the focal subject, their sex, approximate age, reproductive status of the 12 focal subjects included in the study.
**Table S2** Definitions, means and standard deviations ± s.d. or presence absence of events entered into social network analyses. Data based on social behaviour and gestural communication between 132 chimpanzee dyads. All gestural communication measured as the rate per hour dyad spent within 10 m. All social behaviors measured as the rate per hour spent in the same party.
**Table S3** Definitions and descriptive data for variables entered into Generalized Linear Mixed Models, based on 12 chimpanzees. All social behaviours measured as durations (mins), per hour dyad spent in same party.
**Table S4** GLMM results of the association between audience size of partners of same age as the recipient and visual attention.
**Table S5** GLMM results of the association between audience size of partners of same age as the recipient and proximity.
**Table S6** GLMM results of the association between audience size of partners of same age as the signaller and visual attention.
**Table S7** GLMM results of the association between audience size of partners of same age as the signaller and proximity.
**Table S8** GLMM results of the association between total size of party and visual attention.
**Table S9** GLMM results of the association between total size of party and proximity.
**Table S10** GLMM results of the association between response present or absent to a gesture and total number of gesture types in the sequence between dyads who did not reciprocate grooming.
**Table S11** GLMM results of the association between response present or absent to a gesture and total number of manual and bodily gesture types in the sequence between dyads who did not reciprocate grooming.
**Table S12** GLMM results of the association between response present or absent to a gesture and total number of manual (visual, tactile, auditory short range and auditory long range) and bodily (visual, tactile, auditory short range and auditory long range) gesture types in the sequence between dyads who did not reciprocate grooming.
**Table S13** GLMM results of the association between response present or absent to a gesture and total number of gesture types in the sequence between dyads who reciprocated grooming.
**Table S14** GLMM results of the association between response present or absent to a gesture and total number of manual and bodily gesture types in the sequence between dyads who reciprocated grooming.
**Table S15** GLMM results of the association between response present or absent to a gesture and total number of manual (visual, tactile, auditory short range and auditory long range) and bodily (visual, tactile, auditory short range and auditory long range) gesture types in the sequence between dyads who reciprocated grooming.
**Table S16** GLMM results of the association between presence and absence of grooming reciprocity within dyad and social and ecological variables.
**Table S17** GLMM results of the association between total number of gesture types in the sequence and social and ecological variables.
**Table S18** GLMM results of the association between total number of manual gesture types in the sequence and social and ecological variables.
**Table S19** GLMM results of the association between total number of bodily gesture types in the sequence and social and ecological variables.
**Table S20** GLMM results of the association between total number of manual visual gesture types in the sequence and social and ecological variables.
**Table S21** GLMM results of the association between total number of manual tactile gesture types in the sequence and social and ecological variables.
**Table S22** GLMM results of the association between total number of manual auditory long range gesture types in the sequence and social and ecological variables.
**Table S23** GLMM results of the association between total number of bodily visual gesture types in the sequence and social and ecological variables.
**Table S24** GLMM results of the association between total number of bodily auditory long range gesture types in the sequence and social and ecological variables.
**Table S25** GLMM results of the association between total number of bodily auditory short‐range gesture types in the sequence and social and ecological variables
**Table S26** Node‐level regression models predicting durations of social behavior per hour dyad spent in the same party received (indegree). Predictors were repertoire size of gestures produced (outdegree) or received (indegree). Based on 12 chimpanzees. Significant *p* values are indicated in bold.
**Table S27** Node‐level regression models predicting durations of social behavior per hour dyad spent within 10 meters produced (outdegree). Predictors were repertoire size of gestures produced (outdegree) or received (indegree). Based on 12 chimpanzees. Significant *p* values are indicated in bold.
**Table S28** Geary's C statistic predicting durations of social behavior per hour dyad spent in the same party and demography from individual repertoire size attribute (number of gesture types in ones repertoire directed at other adult individuals). Based on 12 chimpanzees. Significant p values are indicated in bold. Smaller values indicate positive autocorrelation whereas a value of 1.0 indicates perfect independence.
**Table S29** Geary's C statistic predicting durations of social behavior per hour dyad spent in the same party from individual repertoire size attribute (number of gesture types directed at other adult individuals). Based on 12 chimpanzees. Significant *p* values are indicated in bold. Smaller values indicate positive autocorrelation whereas a value of 1.0 indicates perfect independence.
