# Supplementary material for: Social and ecological complexity is associated with gestural repertoire size of wild chimpanzees
Source: Integr Zool. 2020 Jun 24;15(4):276–92. doi: 10.1111/1749-4877.12423 (PMC7383666; doi:10.1111/1749-4877.12423)
Supplement: Supplementary file 1 — Table S1 Identity of the focal subject, their sex, approximate age, reproductive status of the 12 focal subjects included in the study. Table S2 Definitions, means and standard deviations ± s.d. or presence absence of events entered into social network analyses. Data based on social behaviour and gestural communication between 132 chimpanzee dyads. All gestural communication measured as the rate per hour dyad spent within 10 m. All social behaviors measured as the rate per hour spent in the same party. Table S3 Definitions and descriptive data for variables entered into Generalized Linear Mixed Models, based on 12 chimpanzees. All social behaviours measured as durations (mins), per hour dyad spent in same party. Table S4 GLMM results of the association between audience size of partners of same age as the recipient and visual attention. Table S5 GLMM results of the association between audience size of partners of same age as the recipient and proximity. Table S6 GLMM results of the association between audience size of partners of same age as the signaller and visual attention. Table S7 GLMM results of the association between audience size of partners of same age as the signaller and proximity. Table S8 GLMM results of the association between total size of party and visual attention. Table S9 GLMM results of the association between total size of party and proximity. Table S10 GLMM results of the association between response present or absent to a gesture and total number of gesture types in the sequence between dyads who did not reciprocate grooming. Table S11 GLMM results of the association between response present or absent to a gesture and total number of manual and bodily gesture types in the sequence between dyads who did not reciprocate grooming. Table S12 GLMM results of the association between response present or absent to a gesture and total number of manual (visual, tactile, auditory short range and auditory long range) and bodily (visual, tactile, auditory [file INZ2-15-276-s001.doc]

**SUPPLEMENTARY MATERIALS**

**Table S1** Identity of the focal subject, their sex, approximate age, reproductive status of the 12 focal subjects included in the study

| **Focal subject ID** | **Sex** | **Age** | **Female reproductive status** | **Total observation duration (hours)** |
| --- | --- | --- | --- | --- |
| BB | Male | 21 | - | 8.60 |
| HW | Male | 15 | - | 17.17 |
| KT | Male | 15 | - | 17.10 |
| KU | Female | 29 | Pregnant | 15.17 |
| KW | Female | 27 | Nursing | 8.50 |
| ML | Female | 33 | Cycling | 18.63 |
| MS | Male | 17 | - | 8.73 |
| NB^b^ | Female | 46 | Cycling | 8.33 |
| NK^a^ | Male | 26 | - | 9.70 |
| RH | Female | 43 | Nursing | 17.30 |
| SQ | Male | 17 | - | 9.23 |
| ZM | Female | 40 | Cycling | 11.83 |

Notes. ^a^ Alpha male^, b^ Alpha female

**Table S2** Definitions, means and standard deviations ± s.d. or presence absence of events entered into social network analyses. Data based on social behaviour and gestural communication between 132 chimpanzee dyads. All gestural communication measured as the rate per hour dyad spent within 10 m. All social behaviors measured as the rate per hour spent in the same party.

| **Gesture** | **Definition** | Mean ± s.d. or presence/ absence |
| --- | --- | --- |
| Sex difference | Sex difference between focal subject and the recipient (0 = different sex, 1 = same sex) | 0 = 60  1 = 72 |
| Age difference | Age difference between focal subject and the recipient (0 = different age, 1 = same age) | 0 = 102  1 = 30 |
| Oestrous difference | Oestrous relationship between focal subject and the recipient: 0 = reproductively inactive (unoestrous female- unoestrous female, unoestrous female-oestrous female, oestrous female-oestrus female, unoestrous female-male, male-male), 1 = reproductively active (male-oestrous female) | 0 = 96  1 = 36 |
| Maternal kinship | Maternal kinship presence between focal subject and the recipient (0 = absent, 1 = present) | 0 = 126  1 = 6 |
| Joint feeding | Duration of jointly feeding when nearest neighbours and within 2 m | 0.62 ± 1.36 |
| Joint resting | Duration of jointly resting when nearest neighbours and within 2 m | 0.99 ± 5.30 |
| Joint travel | Duration of jointly travelling when nearest neighbours and within 2 m | 0.24 ± 0.91 |
| Grooming given | Duration of grooming given to the dyad partner | 0.34 ± 1.09 |
| Grooming received | Duration of grooming received from the dyad partner | 0.32 ± 1.39 |
| Grooming mutual | Duration of mutually grooming with the dyad partner | 0.37 ± 1.63 |
| Attention present | Duration of mutual bodily orientation presence when nearest neighbours and within 2 m of the dyad partner | 1.57 ± 3.66 |
| Attention absent | Duration of mutual bodily orientation absence when nearest neighbours and within 2 meters | 1.84 ± 5.68 |
| Proximity to 2 meters | Duration of proximity when nearest neighbours and within 2 m of the dyad partner | 3.42 ± 7.43 |
| Right-handed | Manual gesture made with a right hand | 0.18 ± 0.60 |
| Left-handed | Manual gesture made with a left hand | 0.41 ± 2.16 |
| Bodily | A gesture is produced by the signaller with the part of the body (e.g. head, legs, torso) that does not involve use of hands | 2.52 ± 6.80 |
| Manual | A gesture is made exclusively with the hand | 0.93 ± 3.19 |
| Non-combined gesture | A single gesture is produced by the signaller without other accompanying gesture type | 2.47 ± 6.60 |
| Combined gesture | Two or more gestures are produced simultaneously by the signaller (e.g. embrace and thrust) | 0.46 ± 1.70 |
| Gesture with no object | Gesture is produced not using object | 2.56 ± 7.14 |
| Gesture with object | Gesture is produced using object (e.g. shake branch) | 0.88 ± 3.12 |
| Events | Number of consecutive gesture events in the sequence. One gesture event can contain gestures combined or not combined with other gestures (e.g. embrace and thrust co-occurring would be counted as one event) | 3.18 ± 7.82 |
| Dyadic repertoire size | The number of gesture types produced towards the dyad partner, per hour spent within 10 m | 1.97 ± 5.11 |
| Indicative | Movement of the arm and hand towards the recipient, without physical touch or contact with substrate | 0.14 ± 0.54 |
| Non-indicative | Movement of the arm and hand that involves physical touch or contact with the substrate or visual but does not involve movement of the hand towards the recipient | 0.80 ± 3.01 |
| Unimodal gesture | Gesture does not include accompanying facial expression or vocalization | 1.79 ± 5.81 |
| Multimodal gesture (facial expression) | Gesture accompanied by simultaneous production of facial expression | 0.09 ± 0.52 |
| Mulitmodal gesture (low amplitude vocalization) | A lower amplitude vocalization other than panthoot is produced whilst the signaller is gesturing | 0.25 ± 0.87 |
| Mulitmodal gesture (high amplitude vocalization) | A high amplitude vocalization such as panthoot call is produced whilst the signaller is gesturing | 0.81 ± 3.25 |
| Gesture with mutual attention absent | Gesture is not accompanied by simultaneous presence of mutual visual contact between signaller and the recipient. Mutual visual contact is when signaller’s and recipient’s body are within each other’s field of view (up to 45 degrees body turn) | 0.78 ± 3.39 |
| Gesture with mutual attention present | Gesture is accompanied by simultaneous presence of mutual visual contact between signaller and the recipient. | 1.23 ± 4.15 |
| Penile erection | Production of a gesture is accompanied by simultaneous erection of the penis by the signaller | 0.19 ± 0.99 |
| Piloerection | Production of a gesture is accompanied by simultaneous involuntary erection of hairs | 1.21 ± 5.11 |
| Non repetitive gesture | A gesture that does not involve repetition of movement in regular and cyclical fashion such as static presentation of a torso for grooming | 1.82 ± 5.72 |
| Repetitive gesture | A gesture involves repetition of movement in regular and cyclical fashion in predictable manner that indicates that the movement forms part of one gesture | 1.69 ± 4.80 |
| Homogenous gesture | Gesture type is present in both signaller’s and recipient’s repertoire of gestures | 1.76 ± 5.49 |
| Heterogeneous gesture | Gesture type occurs only in signaller’s repertoire of gestures | 0.72 ± 2.64 |
| Response present | Presence of any change in the behaviour of the recipient following production of the gesture | 0.94 ± 3.64 |
| Response absent | Absence of any change in the behaviour of the recipient following production of the gesture | 0.58 ± 2.36 |
| Single | Signaller produces one gesture towards the same recipient, with the same goal, within the same context, and made within a maximum of 30 seconds interval. | 1.27 ± 4.07 |
| Rapid sequence | When a chimpanzee produces more than one gesture consecutively within a sequence and there is no intermittent pause between gestures, then the chimpanzee has produced a ‘rapid sequence’. | 0.45 ± 1.30 |
| Persistence sequence | Persistence of gesturing is when the chimpanzee produces one gesture or a gesture sequence, then after a period of response waiting (1-5s) they produce another gesture - here such instances are termed a ‘persistence sequence’. | 0.11 ± 0.45 |
| Close proximity | Signaler produced a gesture within 1 meter from the recipient | 1.09 ± 4.40 |
| Far proximity | Signaler produced a gesture from above 1 meter away from the recipient | 1.64 ± 5.03 |
| Visual gesture | Perception of gesture is only possible by looking at the signaller | 1.97 ± 5.54 |
| Auditory short-range gesture | Sounds produced by the gesture can be heard within 10 m of the signaller | 0.41 ± 2.33 |
| Auditory long-range gesture | Sounds produced by the gesture can be heard over 10 m from the signaller | 0.67 ± 2.30 |
| Tactile gesture | Perception of the gesture is possible via physical contact | 0.44 ± 2.43 |
| Response by visual or tactile gestural communication | Change of behaviour by means of non-vocal behaviour, such as tactile or visual gestures which excludes production of sound by the recipient via vocal tract. This behaviour is not followed by goal directed action towards signaller (e.g. embrace during travel, whereby signallers travel before and after the embrace | 0.08 ± 0.40 |
| Response by activity change | Response to the gesture is by means of goal directed action towards signaller (e.g. approach to groom). | 0.58 ± 1.80 |
| Response by vocalization | Recipient responds to the gesture by means of vocalisation, (production of sound via vocal tract), which is not followed by goal directed action towards signaller (e.g. pantgrunt during travel, whereby signallers travel before and after the pant-grunt | 0.47 ± 2.02 |

**Table S3** Definitions and descriptive data for variables entered into Generalized Linear Mixed Models, based on 12 chimpanzees. All social behaviours measured as durations (mins), per hour dyad spent in same party.

| Behaviour | Definition | Mean ± s.d. or presence/absence |
| --- | --- | --- |
| Overall repertoire size | Total number of all gesture types combined in the single utterance (sequence) | 1.58 ± 1.08 |
| Manual repertoire size | Total number of manual gesture types (produced with the hand or an arm) in the single utterance (sequence) | 0.41 ± 0.60 |
| Bodily repertoire size | Total number of bodily gesture types (produced with any body part other than hand or arm) in the single utterance (sequence) | 1.12 ± 0.82 |
| Manual visual repertoire size | Total number of manual visual gesture types in the single utterance (sequence). Visual gestures can only be received by directing visual attention at the signaller. | 0.11 ± 0.34 |
| Manual auditory short-range repertoire size | Total number of manual, auditory-short range gesture types in the single utterance (sequence). Auditory short-range gestures can be received by hearing over short-distance (e.g. up to approximately 10 meters), where the presence of visual attention is not necessary. | 0.01 ± 0.13 |
| Manual auditory long-range repertoire size | Total number of manual, auditory long-range gesture types in the single utterance (sequence). Auditory long-range gestures can be received by hearing over longer-distance (e.g. further than 10 meters away), where the presence of visual attention is not necessary. | 0.16 ± 0.41 |
| Manual tactile repertoire size | Total number of manual, tactile gesture types in the single utterance (sequence). Tactile gestures can be received through physical contact, where the presence of visual attention is not necessary. | 0.12 ± 0.35 |
| Bodily visual repertoire size | Total number of bodily, visual gesture types in the single utterance (sequence). Visual gestures can only be received by directing visual attention at the signaller. | 0.66 ± 0.72 |
| Bodily auditory short-range repertoire size | Total number of bodily, auditory short-range gesture types in the single utterance (sequence). Auditory short-range gestures can be received by hearing over short-distance (e.g. up to approximately 10 meters), where the presence of visual attention is not necessary. | 0.26 ± 0.44 |
| Bodily auditory long-range repertoire size | Total number of bodily, auditory long-range gesture types in the single utterance (sequence). Auditory long-range gestures can be received by hearing over longer-distance (e.g. further than 10 meters away), where the presence of visual attention is not necessary. | 0.15 ± 0.43 |
| Bodily tactile repertoire size | Total number of bodily, tactile gesture types in the single utterance (sequence). Tactile gestures can be received through physical contact, where the presence of visual attention is not necessary. | 0.04 ± 0.24 |
| Sex difference | Sex difference between focal subject and the recipient (0 = different sex, 1 = same sex) | 0 = 84, 1 = 64 |
| Age difference | Age difference between focal subject and the recipient (0 = different age, 1 = same age) | 0 = 126, 1 = 22 |
| Oestrous difference | Oestrous relationship between focal subject and the recipient: 0 = reproductively inactive (unoestrous female- unoestrous female, unoestrous female-oestrous female, oestrous female-oestrus female, unoestrous female-male, male-male), 1 = reproductively active (male-oestrous female) | 0 = 391, 1 = 122 |
| Maternal kinship | Maternal kinship presence between focal subject and the recipient (0 = absent, 1 = present) | 0 = 142, 1 = 6 |
| Grooming given | Duration of grooming provided by the focal subject to the dyad partner | 1.42 ± 2.30 |
| Grooming received | Duration of grooming received by the focal subject from the non-focal dyad partner | 1.08 ± 2.33 |
| Grooming mutual | Duration of mutually grooming with the dyad partner | 1.26 ± 2.64 |
| Proximity | Focal and non-focal subjects are within 2 meters of one another | 9.79 ± 9.55 |
| Attention present | Both focal and non-focal subjects are bodily oriented towards one another (one has another within field of view up to 45 degrees body turn) | 6.03 ± 7.86 |
| Attention absent | Both focal and non-focal subjects are bodily oriented away from one another (one has another away from field of view up to 45 degrees body turn) | 3.81 ± 3.48 |
| Joint feeding | Focal and non-focal subject consume food simultaneously | 1.26 ± 1.86 |
| Joint resting | Focal and non-focal subject are in resting position not involved in any activity | 1.90 ± 2.25 |
| Joint travel | Focal and non-focal subjects simultaneously relocate from one location in the habitat to another | 0.77 ± 1.58 |
| Party size | Total number of individuals in the party | 7.51 ± 5.44 |
| Audience same age as focal | Number of individuals in the audience who are of the same age as the focal subject who is producing a gesture | 0.32 ± 0.63 |
| Audience same age as recipient | Number of individuals in the audience who are of the same age as the recipient of the gesture | 0.26 ± 0.63 |
| Recipient proximity | Distance to recipient during production of the gesture (m) | 2.41 ± 4.27 |
| Reciprocity | Bouts of giving grooming between focal subject and non-focal subject are either 0 - non-reciprocated (non-focal subject who is the recipient of unidirectional grooming does not reciprocate by grooming unidirectionally or mutually grooming), 1 – reciprocated (non-focal subject who is the recipient of grooming grooms back the focal subject who is the groomee either unidirectionally or by engaging in mutual grooming) | 0 = 174, 1 = 159 |
| Body orientation | Orientation of the body of the recipient relative to a signaller prior to production of first gesture: recipient facing signaller with the back (0), recipient facing signaller with the chest or side of the body (1) | 0 = 68, 1 = 351 |
| Response | Response absent (0) or present (1) | 0 = 208, 1 = 258 |
| Noise | Noise level (dB) | 59.59 ± 21.05 |
| Illumination | Illumination level (lx) | 1245.98 ± 2016.48 |
| Temperature | Temperature (°C) | 25.76 ± 2.57 |
| Visibility | Visibility in meters | 20.46 ± 6.14 |
| Wind | Wind (meters per second) | 0.01 ± 0.10 |
| Visitor number | Number of human visitors attending to a party | 3.73 ± 1.48 |
| Visitor distance | Distance between focal subject and the nearest human visitor in the audience | 14.07 ± 9.46 |

**Table S4** GLMM results of the association between audience size of partners of same age as the recipient and visual attention


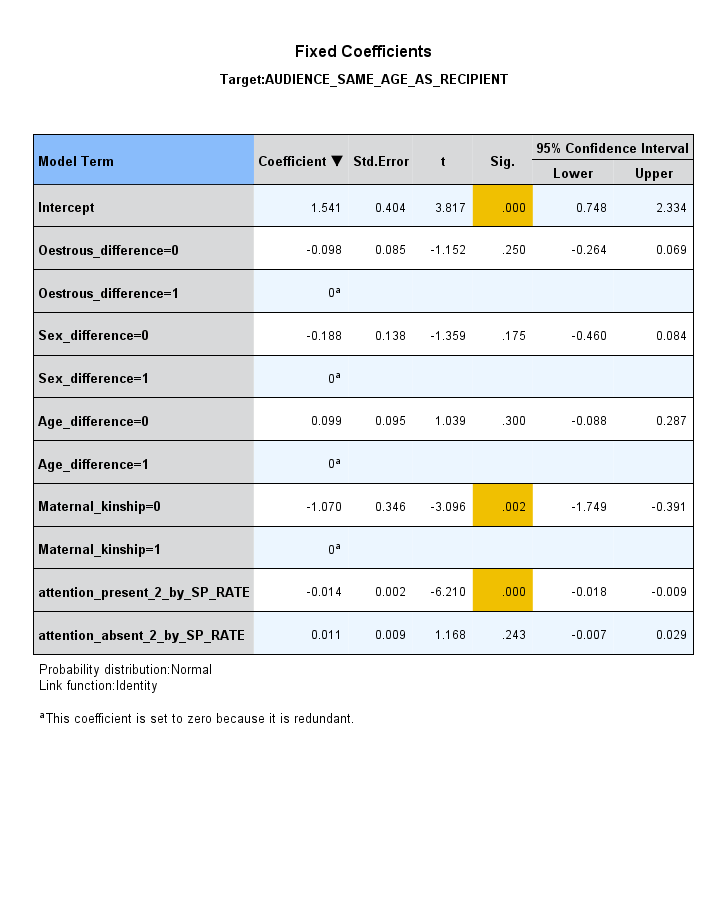


**Table S5** GLMM results of the association between audience size of partners of same age as the recipient and proximity


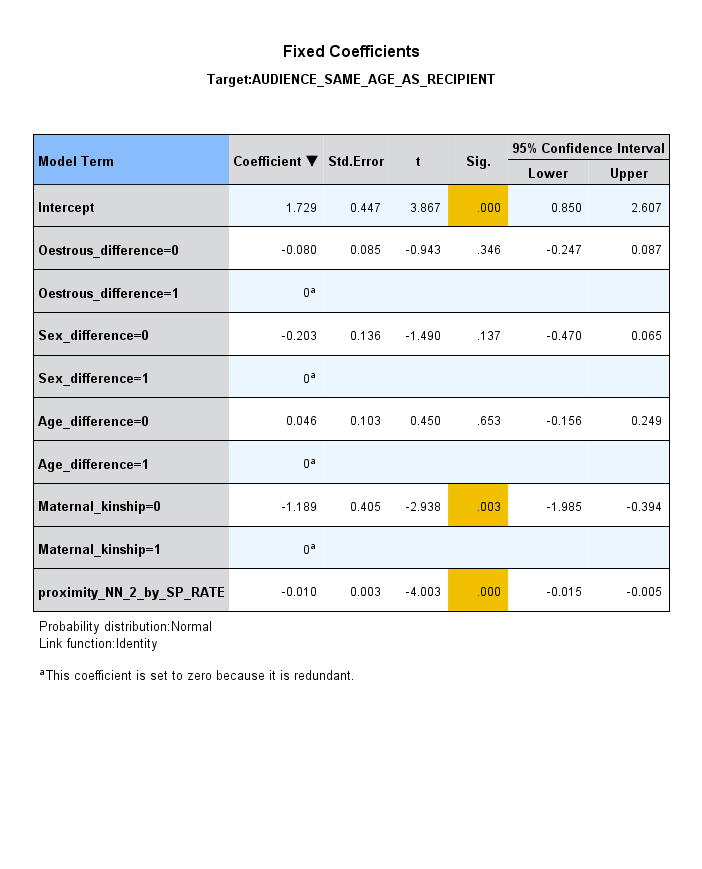


**Table S6** GLMM results of the association between audience size of partners of same age as the signaller and visual attention


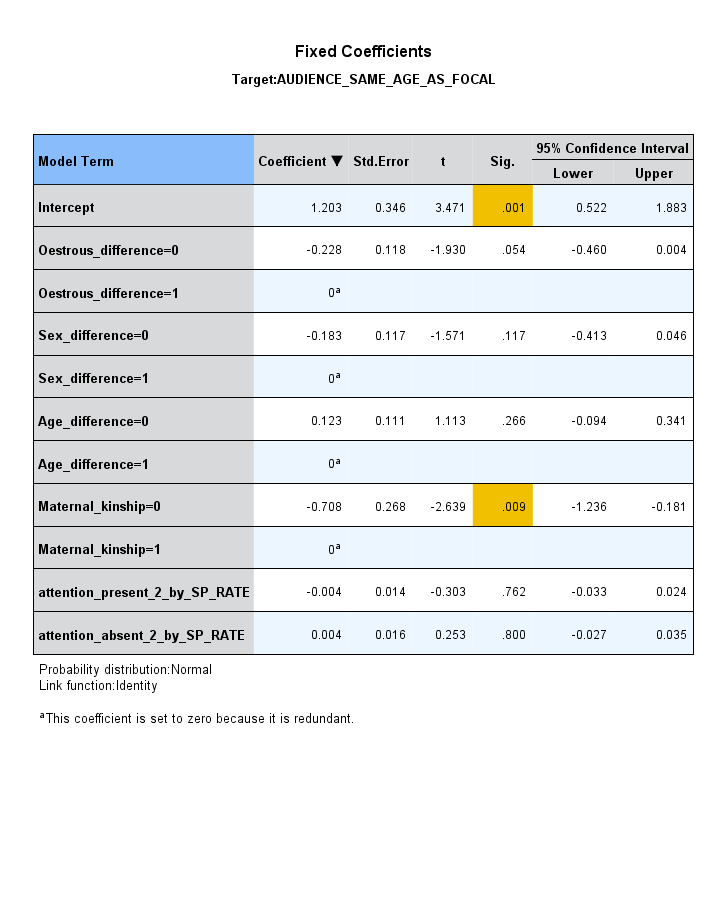


**Table S7** GLMM results of the association between audience size of partners of same age as the signaller and proximity


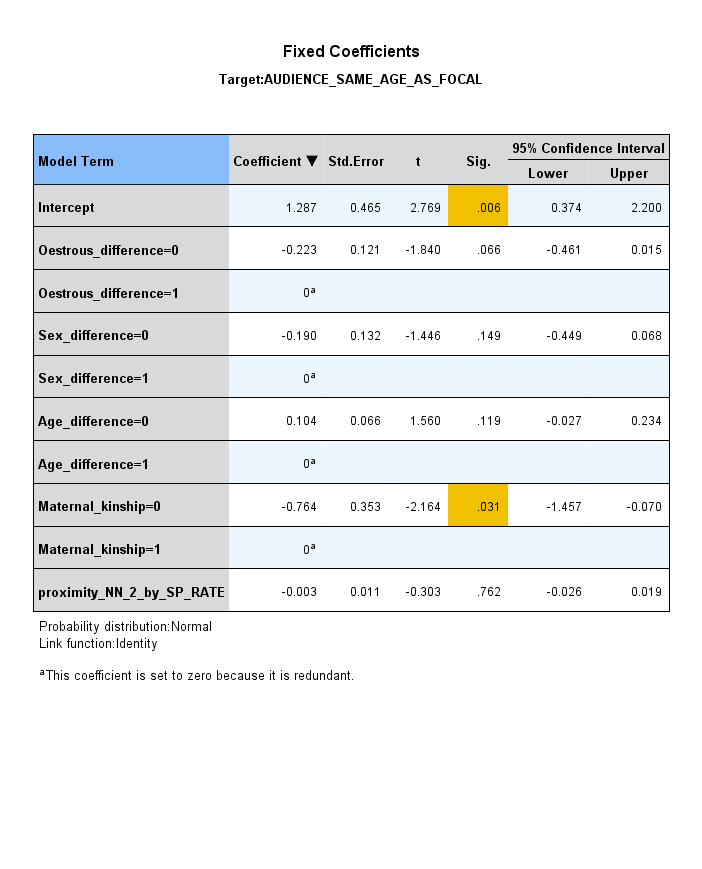


**Table S8** GLMM results of the association between total size of party and visual attention


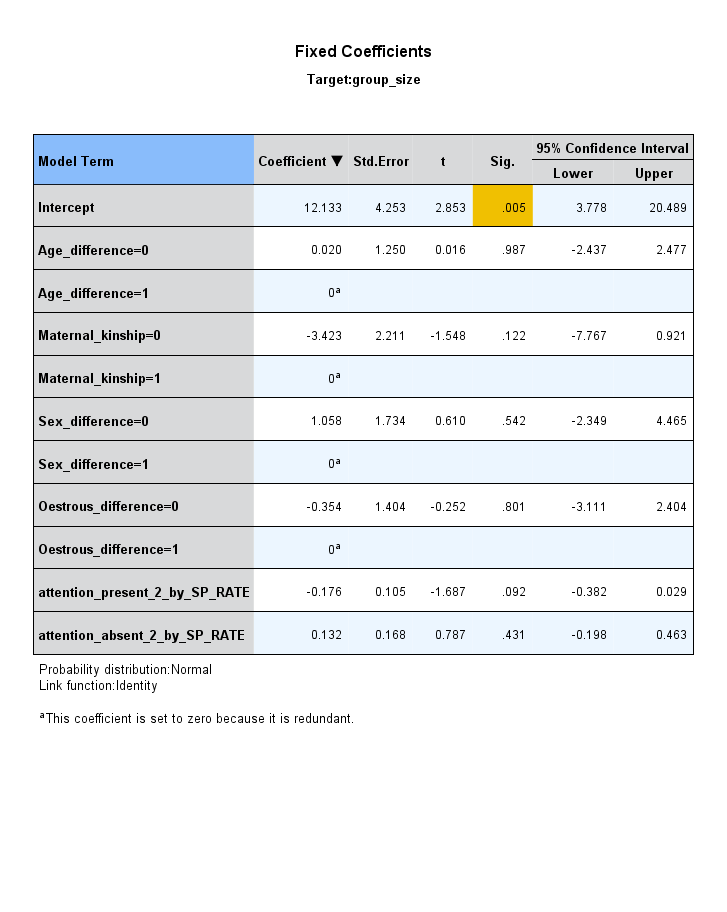


**Table S9** GLMM results of the association between total size of party and proximity


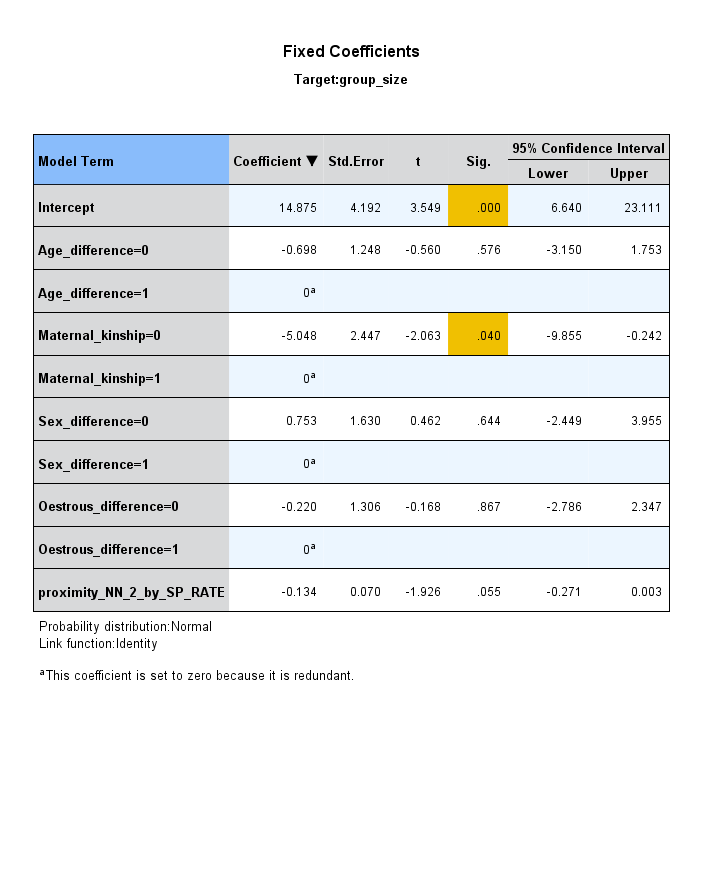


**Table S10** GLMM results of the association between response present or absent to a gesture and total number of gesture types in the sequence between dyads who did not reciprocate grooming


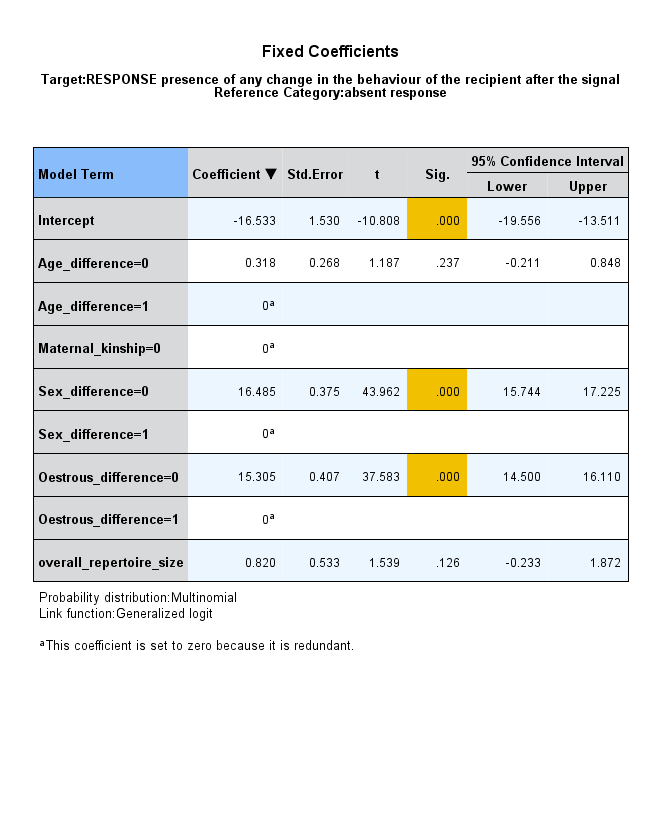


**Table S11** GLMM results of the association between response present or absent to a gesture and total number of manual and bodily gesture types in the sequence between dyads who did not reciprocate grooming


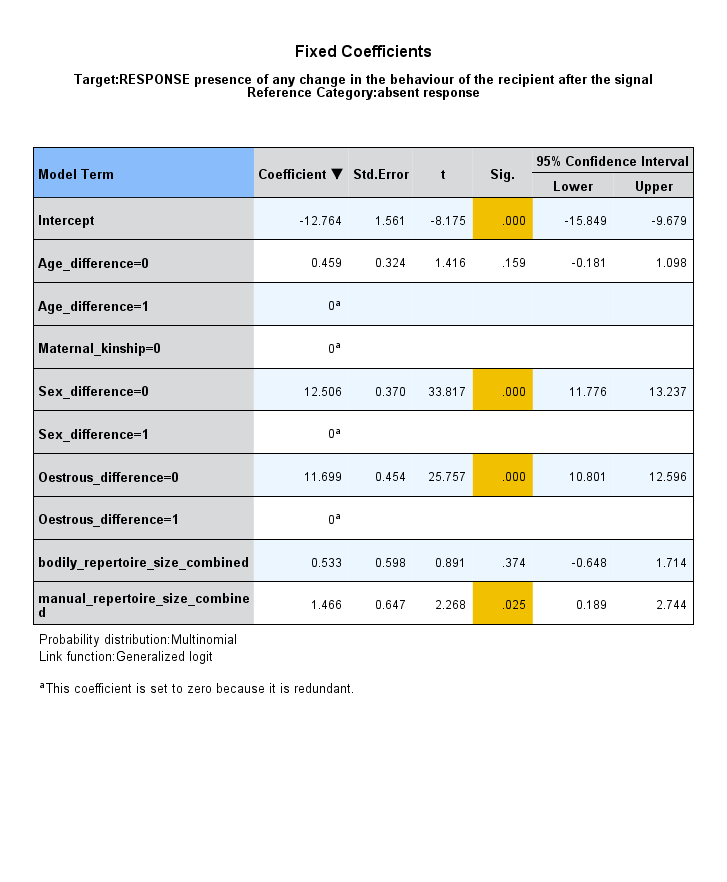


**Table S12** GLMM results of the association between response present or absent to a gesture and total number of manual (visual, tactile, auditory short range and auditory long range) and bodily (visual, tactile, auditory short range and auditory long range) gesture types in the sequence between dyads who did not reciprocate grooming


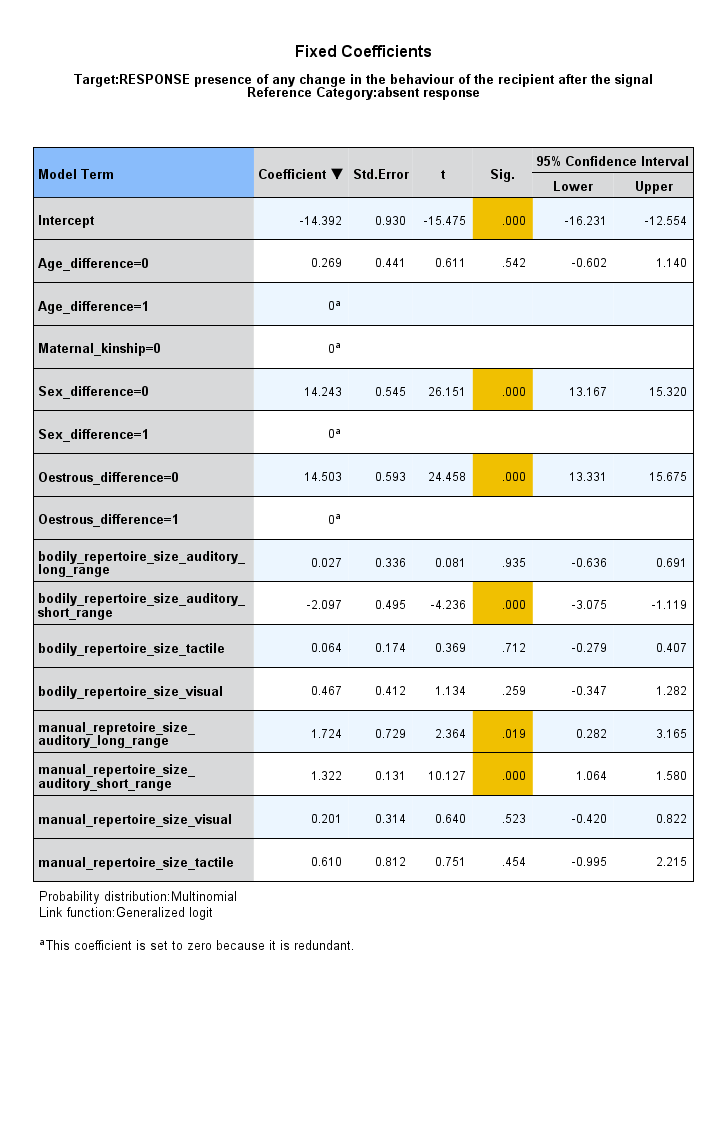


**Table S13** GLMM results of the association between response present or absent to a gesture and total number of gesture types in the sequence between dyads who reciprocated grooming


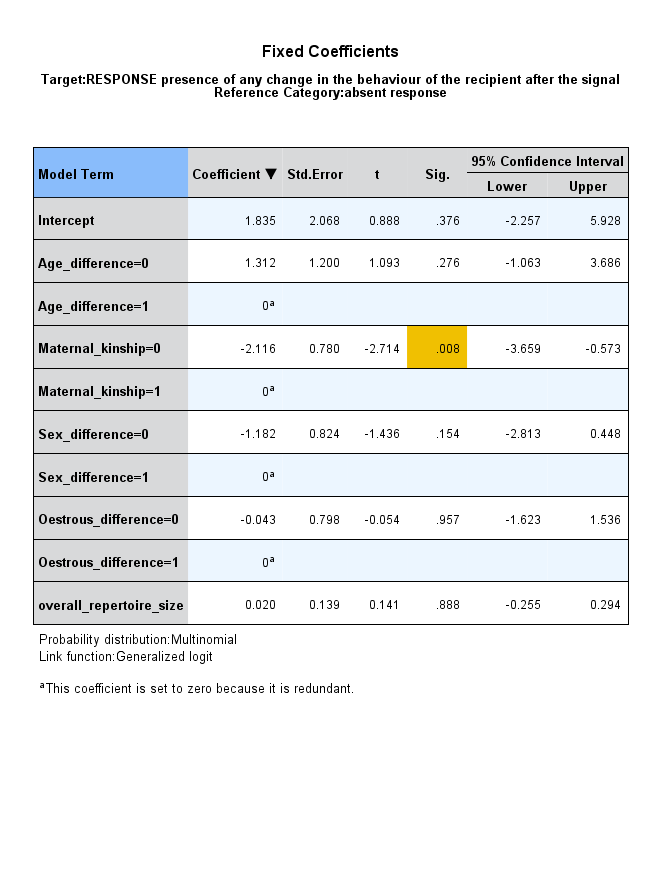


**Table S14** GLMM results of the association between response present or absent to a gesture and total number of manual and bodily gesture types in the sequence between dyads who reciprocated grooming


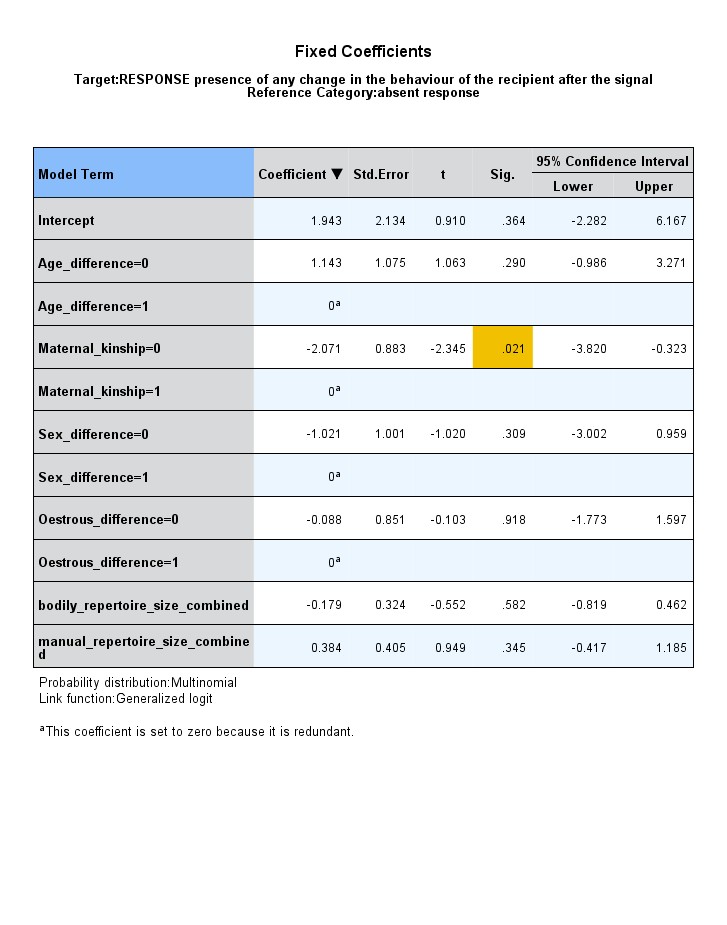


**Table S15** GLMM results of the association between response present or absent to a gesture and total number of manual (visual, tactile, auditory short range and auditory long range) and bodily (visual, tactile, auditory short range and auditory long range) gesture types in the sequence between dyads who reciprocated grooming


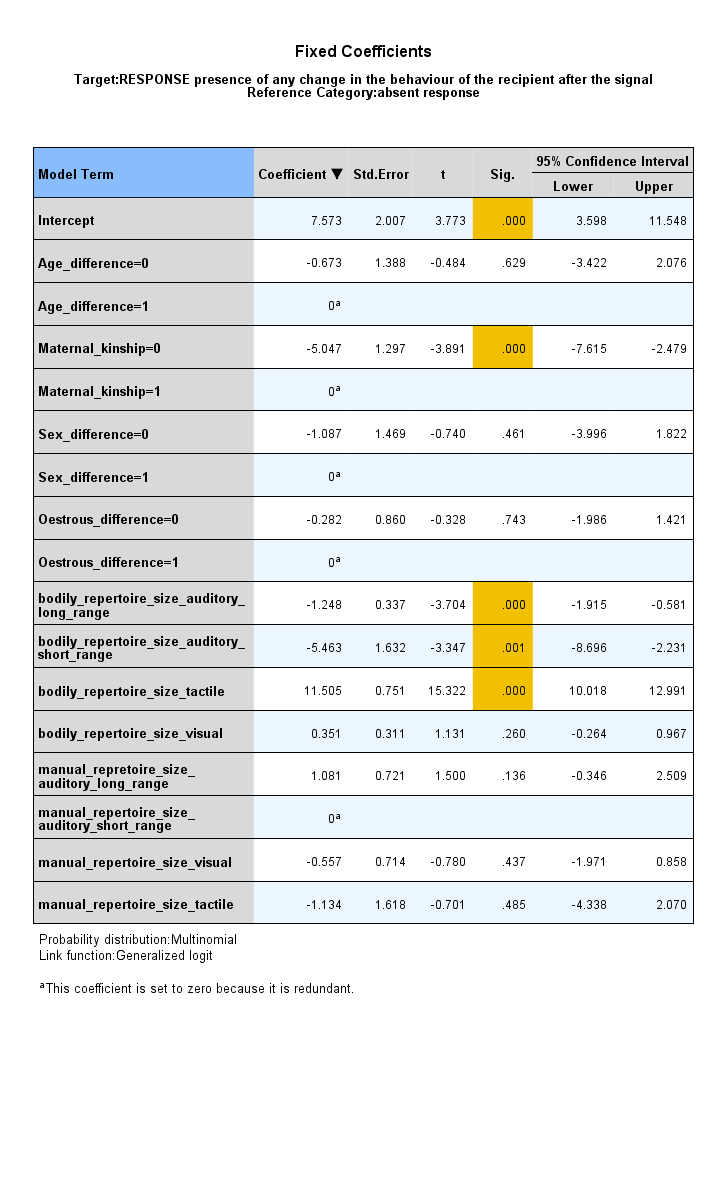


**Table S16** GLMM results of the association between presence and absence of grooming reciprocity within dyad and social and ecological variables


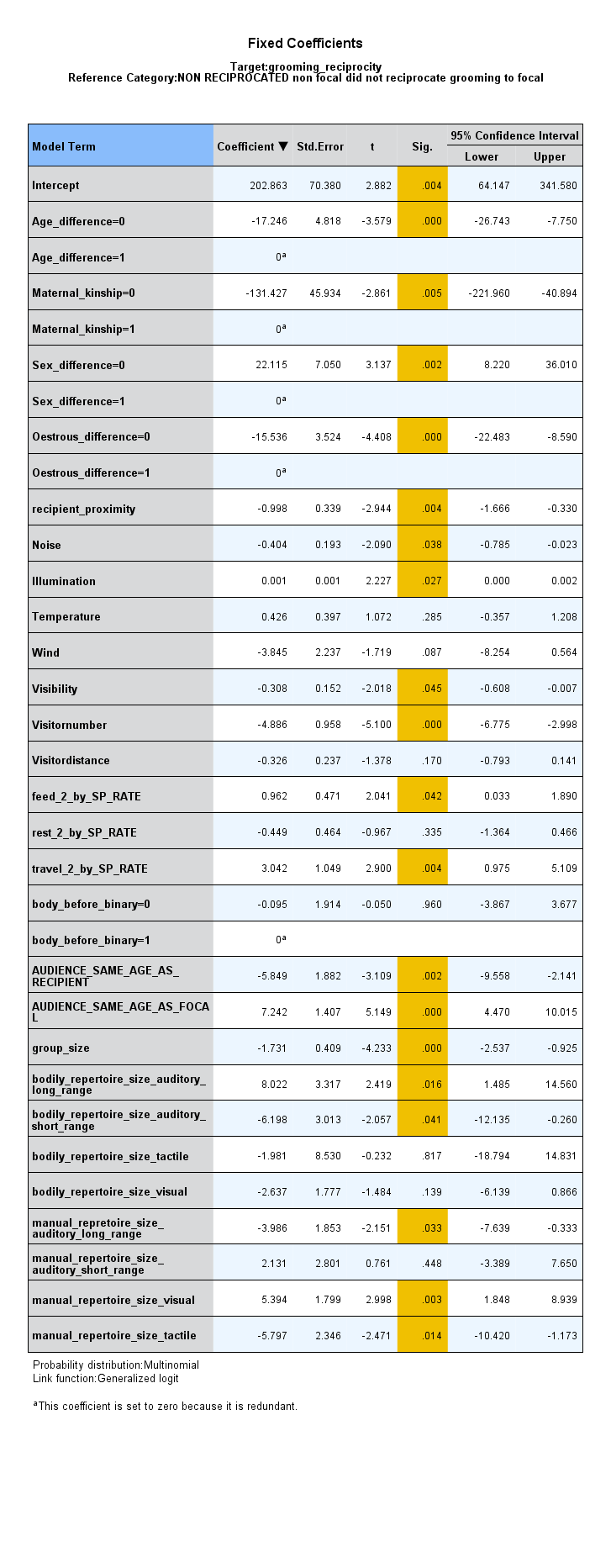


**Table S17** GLMM results of the association between total number of gesture types in the sequence and social and ecological variables


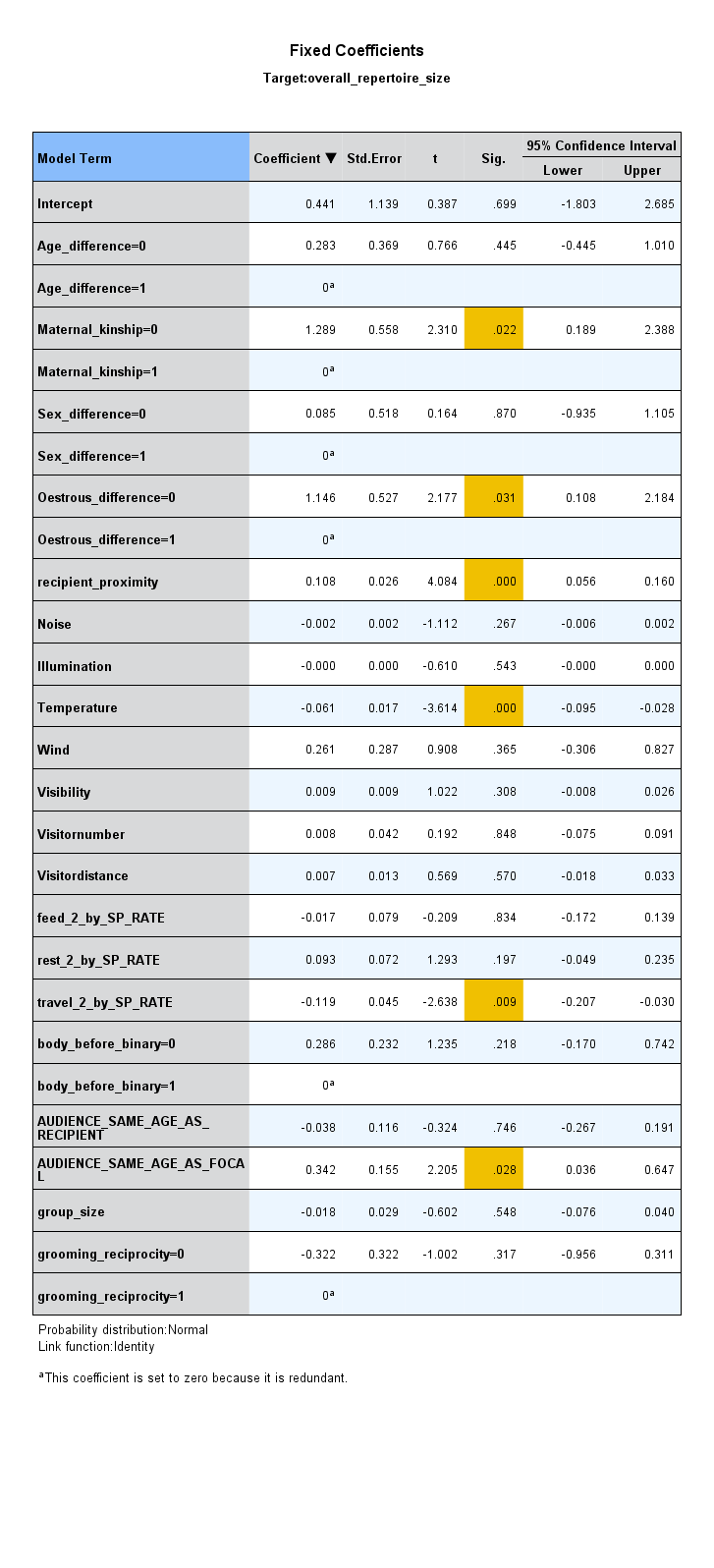


**Table S18** GLMM results of the association between total number of manual gesture types in the sequence and social and ecological variables


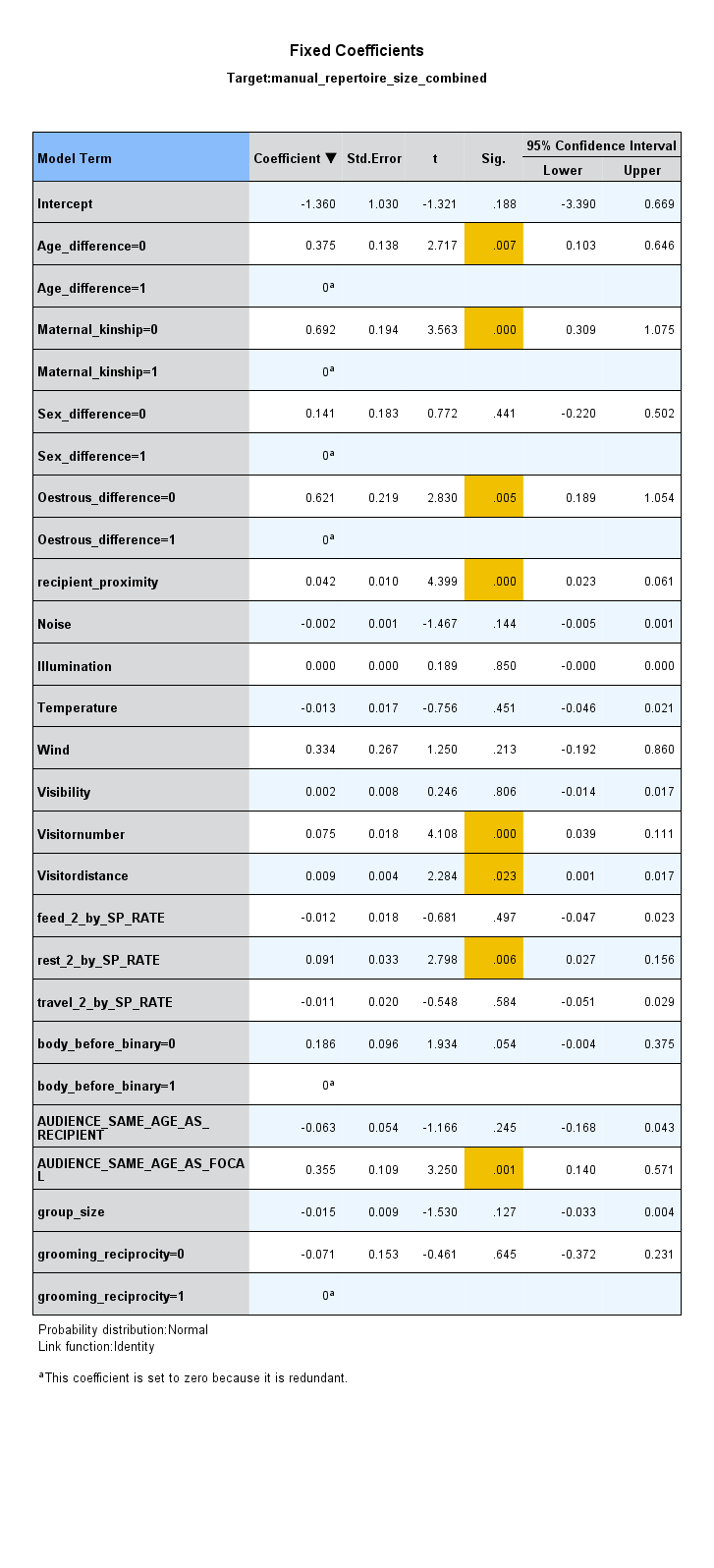


**Table S19** GLMM results of the association between total number of bodily gesture types in the sequence and social and ecological variables


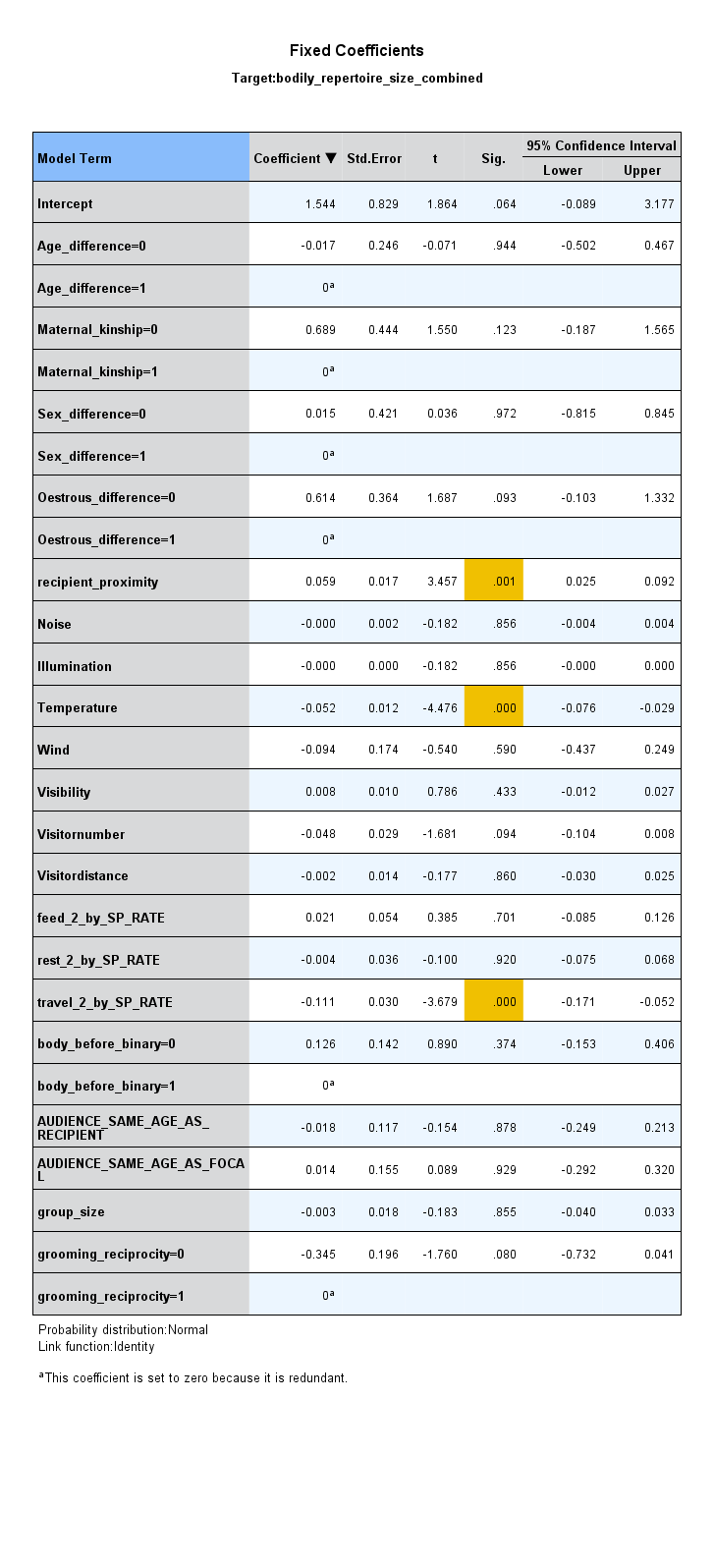


**Table S20** GLMM results of the association between total number of manual visual gesture types in the sequence and social and ecological variables


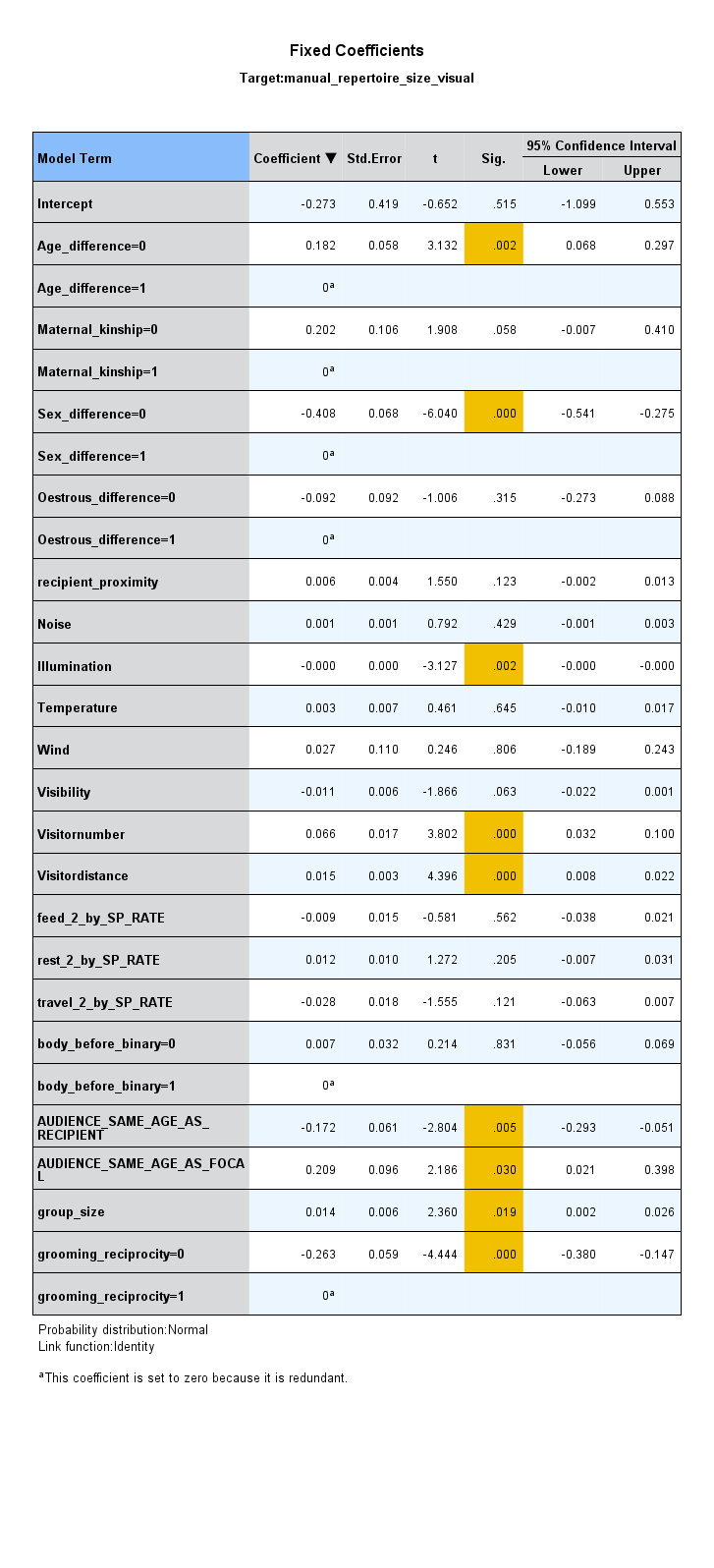


**Table S21** GLMM results of the association between total number of manual tactile gesture types in the sequence and social and ecological variables


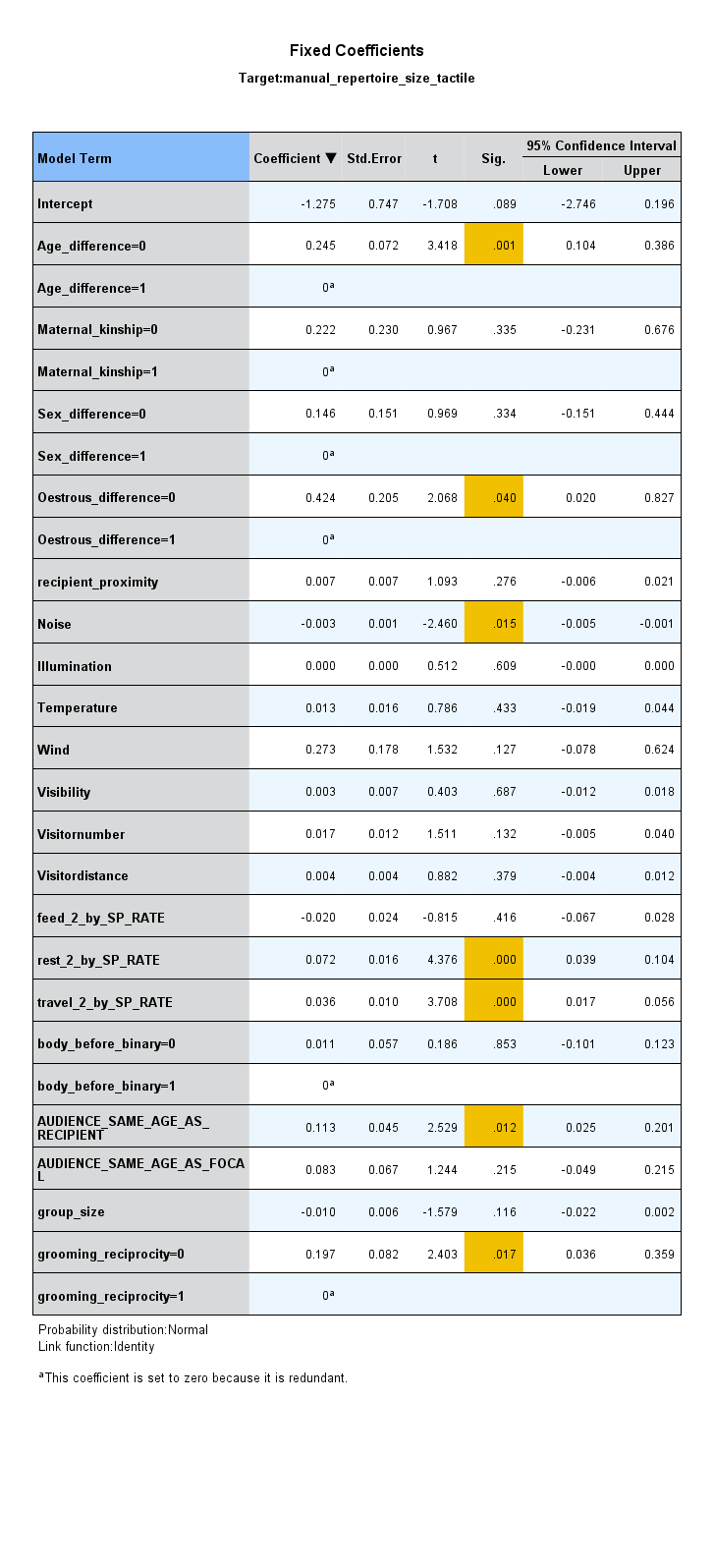


**Table S22** GLMM results of the association between total number of manual auditory long range gesture types in the sequence and social and ecological variables


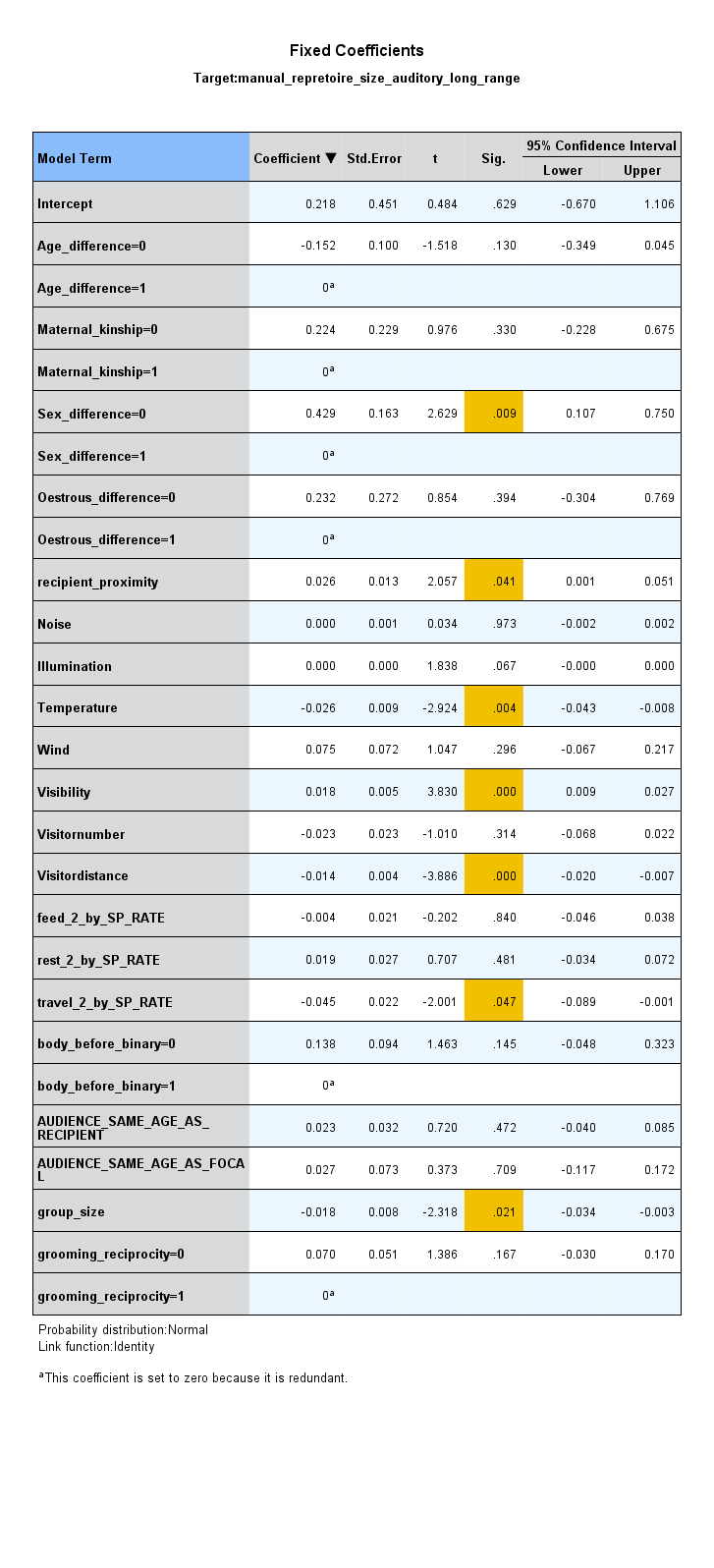


**Table 23** GLMM results of the association between total number of bodily visual gesture types in the sequence and social and ecological variables


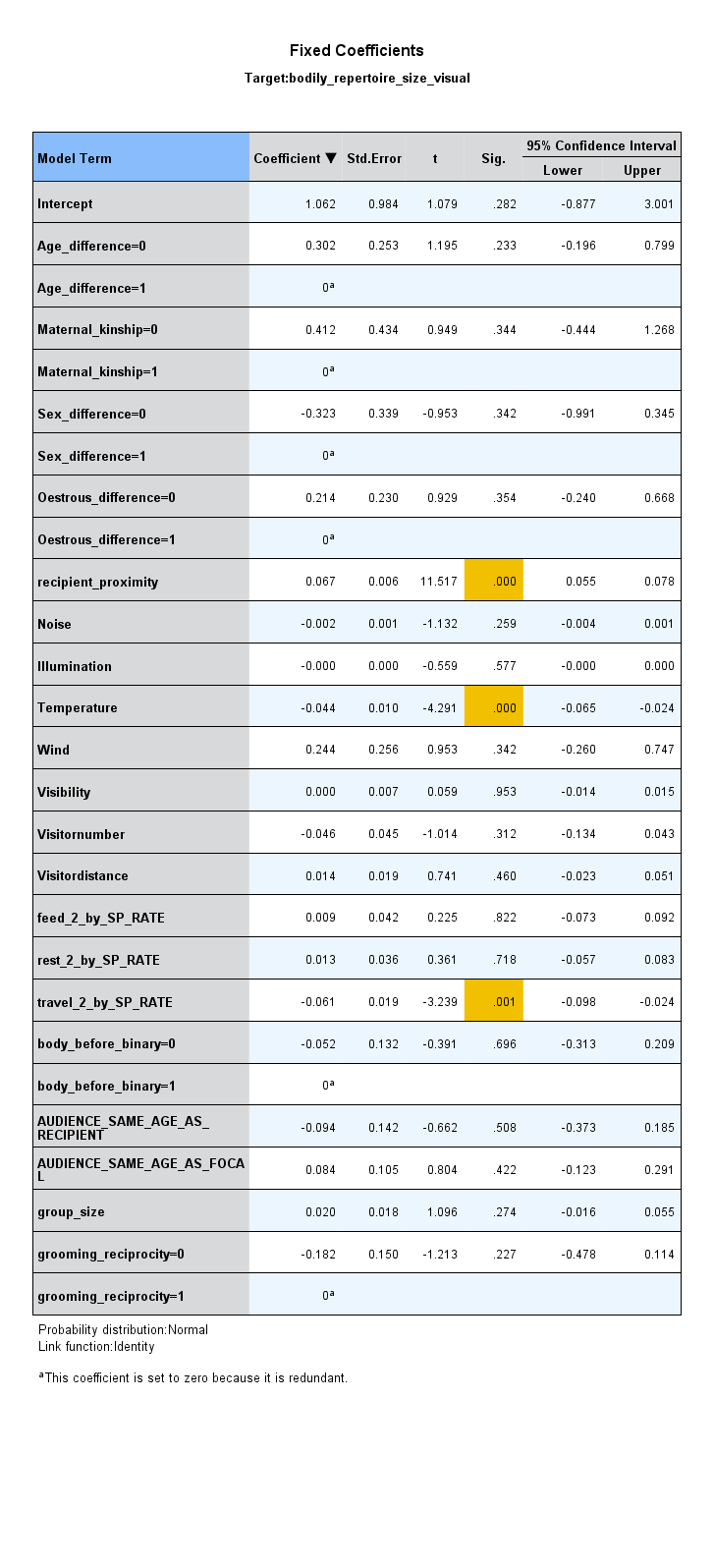


**Table S24** GLMM results of the association between total number of bodily auditory long range gesture types in the sequence and social and ecological variables


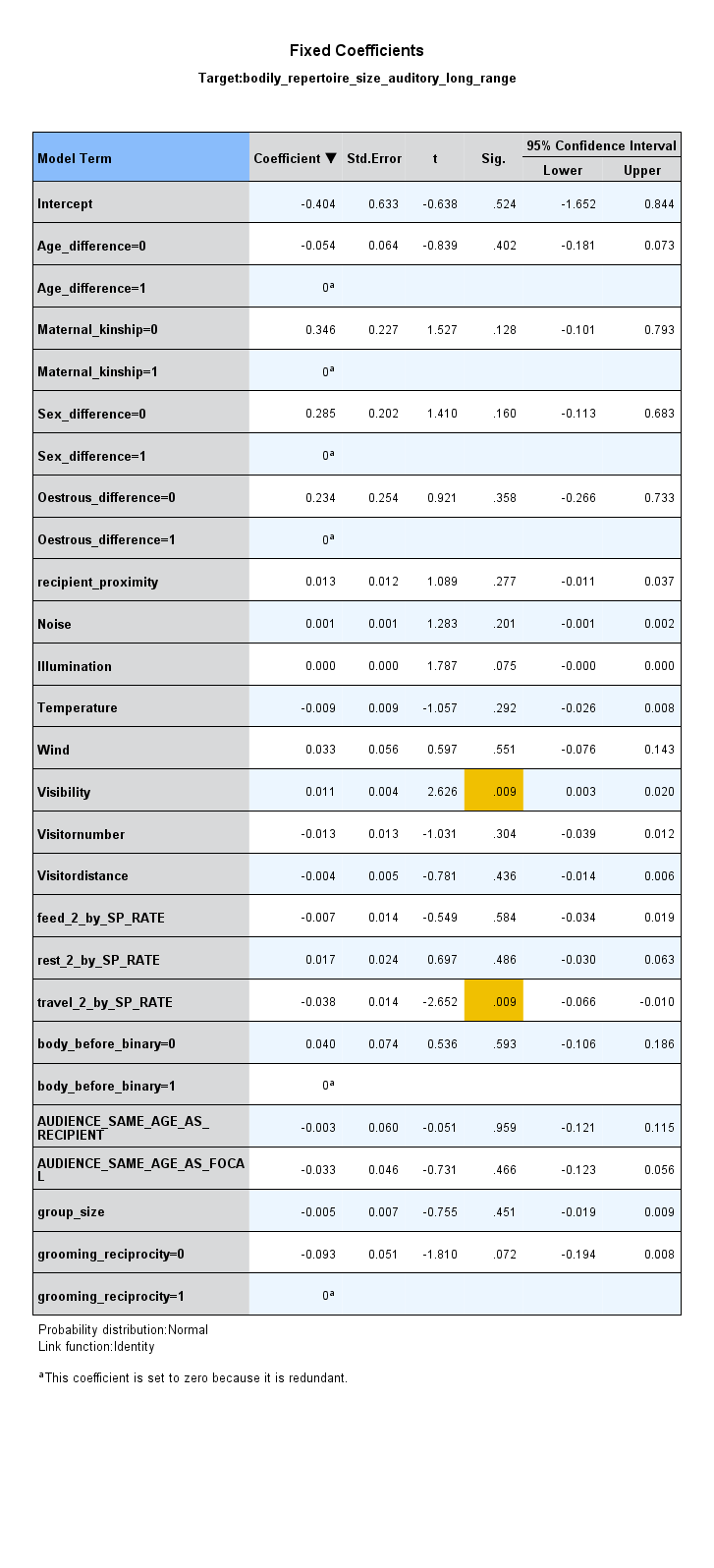


**Table S25** GLMM results of the association between total number of bodily auditory short-range gesture types in the sequence and social and ecological variables


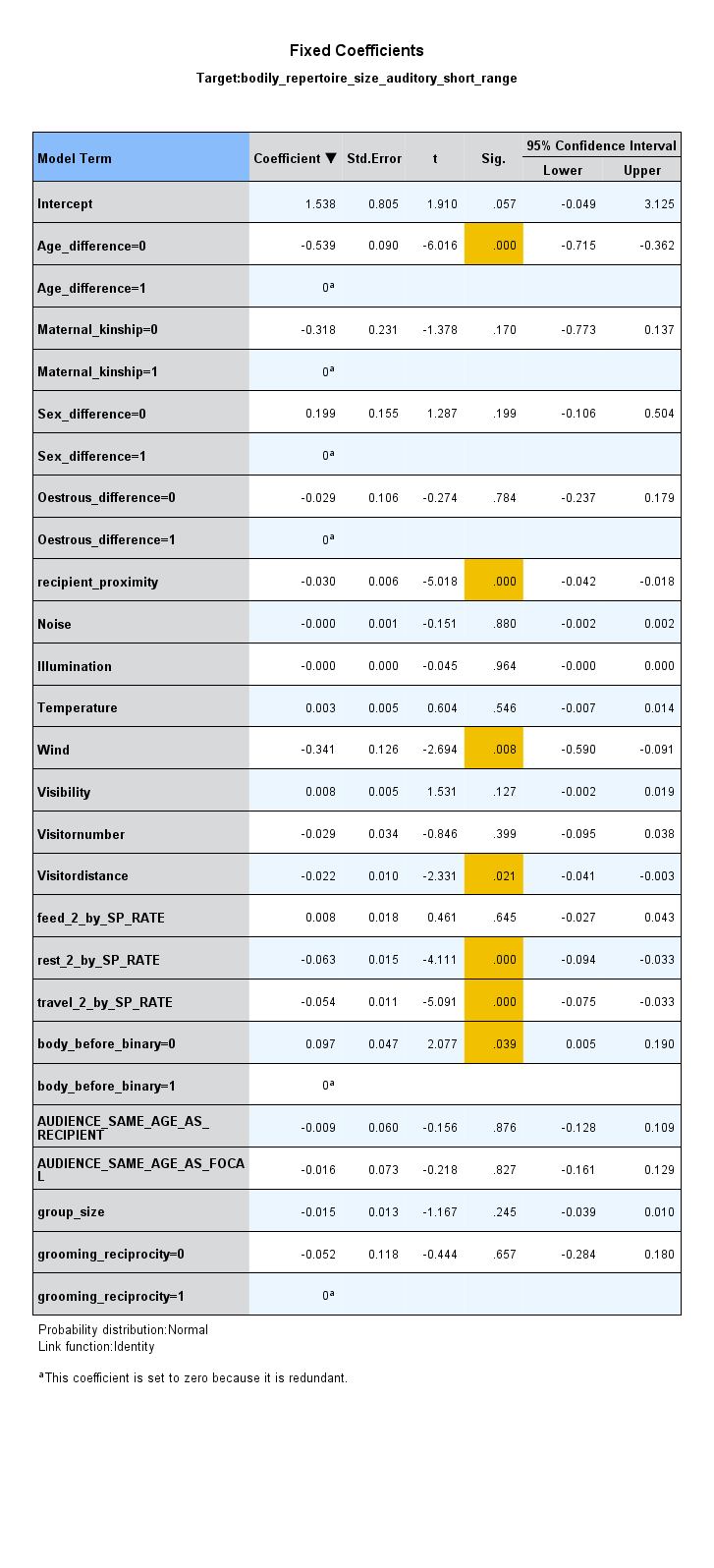


GESTURES AND SOCIALITY CENTRALITY INDEGREE

**Table S26** Node-level regression models predicting durations of social behavior per hour dyad spent in the same party received (indegree). Predictors were repertoire size of gestures produced (outdegree) or received (indegree). Based on 12 chimpanzees. Significant *p* values are indicated in bold.

**Table S26.1** Duration of proximity to 2 meters indegree

| Attribute category/ Agreement in gesture repertoires | Standardized coefficient | *P* |
| --- | --- | --- |
| r^2^ = 0.466 | | |
| Reproductive state of female | –0.066 | 0.494 |
| Kinship | 0.597 | 0.098 |
| Sex/age | 0.344 | 0.240 |
| Repertoire size outdegree | 0.099 | 0.417 |
| Repertoire size indegree | 0.672 | **0.047** |

**Table S26.2** Duration of joint feeding indegree

| Attribute category/ Agreement in gesture repertoires | Standardized coefficient | *P* |
| --- | --- | --- |
| r^2^ = 0.434 | | |
| Reproductive state of female | 0.388 | 0.138 |
| Kinship | 0.231 | 0.316 |
| Sex/ age | –0.071 | 0.431 |
| Repertoire size outdegree | 0.542 | 0.133 |
| Repertoire size indegree | 0.331 | 0.220 |

**Table S26.3** Duration of joint resting indegree

| Attribute category/ Agreement in gesture repertoires | Standardized coefficient | *P* |
| --- | --- | --- |
| r^2^ = 0.429 | | |
| Reproductive state of female | –0.255 | 0.226 |
| Kinship | 0.688 | 0.063 |
| Sex/ age | 0.800 | 0.043 |
| Repertoire size outdegree | –0.523 | 0.129 |
| Repertoire size indegree | 0.317 | 0.215 |

**Table S26.4 Duration of joint travelling indegree**

| Attribute category/ Agreement in gesture repertoires | Standardized coefficient | *P* |
| --- | --- | --- |
| r^2^ = 0.638 | | |
| Reproductive state of female | –0.303 | 0.195 |
| Kinship | 0.102 | 0.425 |
| Sex/ age | 0.218 | 0.320 |
| Repertoire size outdegree | 0.065 | 0.436 |
| Repertoire size indegree | 0.738 | **0.031** |

**Table S26.5 Duration of mutual attention present indegree**

| Attribute category/ Agreement in gesture repertoires | Standardized coefficient | *P* |
| --- | --- | --- |
| r^2^ = 0.732 | | |
| Reproductive state of female | 0.124 | 0.313 |
| Kinship | 0.298 | 0.271 |
| Sex/ age | –0.264 | 0.293 |
| Repertoire size outdegree | 0.485 | 0.148 |
| Repertoire size indegree | 0.667 | **0.048** |

**Table S26.6 Duration of mutual attention absent indegree**

| Attribute category/ Agreement in gesture repertoires | Standardized coefficient | *P* |
| --- | --- | --- |
| r^2^ = 0.396 | | |
| Reproductive state of female | –0.228 | 0.285 |
| Kinship | 0.641 | 0.079 |
| Sex/ age | 0.802 | **0.036** |
| Repertoire size outdegree | –0.328 | 0.247 |
| Repertoire size indegree | 0.393 | 0.169 |

**Table S26.7** Duration of grooming given indegree

| Attribute category/ Agreement in gesture repertoires | Standardized coefficient | *P* |
| --- | --- | --- |
| r^2^ = 0.682 | | |
| Reproductive state of female | 0.003 | 0.391 |
| Kinship | 0.340 | 0.240 |
| Sex/ age | –0.214 | 0.328 |
| Repertoire size outdegree | 0.442 | 0.168 |
| Repertoire size indegree | 0.653 | **0.047** |

**Table S26.8** Duration of grooming received indegree

| Attribute category/ Agreement in gesture repertoires | Standardized coefficient | *P* |
| --- | --- | --- |
| r^2^ = 0.108 | | |
| Reproductive state of female | –0.212 | 0.309 |
| Kinship | –0.129 | 0.393 |
| Sex/ age | 0.105 | 0.414 |
| Repertoire size outdegree | 0.035 | 0.469 |
| Repertoire size indegree | 0.103 | 0.396 |

**Table S26.9** Duration of grooming mutual indegree

| Attribute category/ Agreement in gesture repertoires | Standardized coefficient | *P* |
| --- | --- | --- |
| r^2^ = 0.820 | | |
| Reproductive state of female | –0.061 | 0.510 |
| Kinship | 0.219 | 0.334 |
| Sex/ age | –0.238 | 0.292 |
| Repertoire size outdegree | 0.354 | 0.234 |
| Repertoire size indegree | 0.794 | **0.026** |

GESTURES AND SOCIALITY CENTRALITY OUTDEGREE

**Table S27** Node-level regression models predicting durations of social behavior per hour dyad spent within 10 meters produced (outdegree). Predictors were repertoire size of gestures produced (outdegree) or received (indegree). Based on 12 chimpanzees. Significant *p* values are indicated in bold.

**Table S27.1** Duration of proximity to 2 meters outdegree

| Attribute category/ Agreement in gesture repertoires | Standardized coefficient | *P* |
| --- | --- | --- |
| r^2^ = 0.680 | | |
| Reproductive state of female | 0.278 | 0.153 |
| Kinship | 0.409 | 0.190 |
| Sex/ age | –0.308 | 0.237 |
| Repertoire size outdegree | 0.764 | **0.047** |
| Repertoire size indegree | 0.323 | 0.214 |

**Table S27.2** Duration of joint feeding outdegree

| Attribute category/ Agreement in gesture repertoires | Standardized coefficient | *P* |
| --- | --- | --- |
| r^2^ = 0.580 | | |
| Reproductive state of female | 0.402 | 0.110 |
| Kinship | 0.416 | 0.199 |
| Sex/ age | –0.497 | 0.147 |
| Repertoire size outdegree | 0.280 | 0.280 |
| Repertoire size indegree | –0.074 | 0.439 |

**Table S27.3** Duration of joint resting outdegree

| Attribute category/ Agreement in gesture repertoires | Standardized coefficient | *P* |
| --- | --- | --- |
| r^2^ = 0.530 | | |
| Reproductive state of female | 0.289 | 0.113 |
| Kinship | 0.323 | 0.264 |
| Sex/ age | –0.253 | 0.249 |
| Repertoire size outdegree | 0.756 | **0.047** |
| Repertoire size indegree | 0.188 | 0.279 |

**Table S27.4** Duration of joint travelling outdegree

| Attribute category/ Agreement in gesture repertoires | Standardized coefficient | *P* |
| --- | --- | --- |
| r^2^ = 0.715 | | |
| Reproductive state of female | 0.321 | 0.158 |
| Kinship | 0.361 | 0.226 |
| Sex/ age | –0.162 | 0.357 |
| Repertoire size outdegree | 0.683 | 0.072 |
| Repertoire size indegree | 0.493 | 0.120 |

**Table S27.5** Duration of mutual attention present outdegree

| Attribute category/ Agreement in gesture repertoires | Standardized coefficient | *P* |
| --- | --- | --- |
| r^2^ = 0.681 | | |
| Reproductive state of female | 0.289 | 0.172 |
| Kinship | 0.379 | 0.216 |
| Sex/ age | –0.386 | 0.207 |
| Repertoire size outdegree | 0.697 | 0.068 |
| Repertoire size indegree | 0.385 | 0.180 |

**Table S27.6** Duration of mutual attention absent outdegree

| Attribute category/ Agreement in gesture repertoires | Standardized coefficient | *P* |
| --- | --- | --- |
| r^2^ = 0.670 | | |
| Reproductive state of female | 0.267 | 0.144 |
| Kinship | 0.419 | 0.188 |
| Sex/ age | –0.255 | 0.266 |
| Repertoire size outdegree | 0.791 | **0.037** |
| Repertoire size indegree | 0.279 | 0.236 |

**Table S27.7** Duration of grooming given outdegree

| Attribute category/ Agreement in gesture repertoires | Standardized coefficient | *P* |
| --- | --- | --- |
| r^2^ = 0.381 | | |
| Reproductive state of female | –0.03 | 0.51 |
| Kinship | 0.19 | 0.35 |
| Sex/ age | –0.05 | 0.47 |
| Repertoire size outdegree | 0.63 | 0.08 |
| Repertoire size indegree | –0.09 | 0.41 |

**Table S27.8** Duration of grooming received outdegree

| Attribute category/ Agreement in gesture repertoires | Standardized coefficient | *P* |
| --- | --- | --- |
| r^2^ = 0.707 | | |
| Reproductive state of female | 0.071 | 0.309 |
| Kinship | 0.311 | 0.273 |
| Sex/ age | –0.303 | 0.243 |
| Repertoire size outdegree | 0.622 | 0.093 |
| Repertoire size indegree | 0.516 | 0.103 |

**Table S27.9** Duration of grooming mutual outdegree

| Attribute category/ Agreement in gesture repertoires | Standardized coefficient | *P* |
| --- | --- | --- |
| r^2^ = 0.650 | | |
| Reproductive state of female | 0.323 | 0.159 |
| Kinship | 0.270 | 0.292 |
| Sex/ age | –0.282 | 0.272 |
| Repertoire size outdegree | 0.574 | 0.111 |
| Repertoire size indegree | 0.521 | 0.109 |

GESTURES AND SOCIAL HOMOPHILLY

**Table S28** Geary’s C statistic predicting durations of social behavior per hour dyad spent in the same party and demography from individual repertoire size attribute (number of gesture types in ones repertoire directed at other adult individuals). Based on 12 chimpanzees. Significant *p* values are indicated in bold. Smaller values indicate positive autocorrelation whereas a value of 1.0 indicates perfect independence

| Dependent variable | Geary’s C statistic | *P* value |
| --- | --- | --- |
| Age similarity | 0.408 | **0.020** |
| Sex similarity | 0.167 | **0.002** |
| Kinship | 2.208 | **0.021** |
| Oestrus similarity | 1.472 | **0.036** |
| Joint rest | 1.616 | 0.131 |
| Joint travel | 0.425 | **0.035** |
| Joint feed | 0.768 | 0.177 |
| Attention absent | 1.178 | 0.244 |
| Attention present | 0.499 | **0.006** |
| Proximity | 0.865 | 0.318 |
| Groom given | 0.474 | **0.036** |
| Groom mutual | 0.382 | **0.048** |
| Groom received | 0.400 | **0.022** |

GESTURES AND COMMUNICATIVE COMPLEXITY HOMOPHILLY

**Table S29** Geary’s C statistic predicting durations of social behavior per hour dyad spent in the same party from individual repertoire size attribute (number of gesture types directed at other adult individuals). Based on 12 chimpanzees. Significant *p* values are indicated in bold. Smaller values indicate positive autocorrelation whereas a value of 1.0 indicates perfect independence

| Dependent variable | Geary’s C statistic | *P* value |
| --- | --- | --- |
| Bodily | 0.498 | **0.011** |
| Manual | 0.649 | 0.135 |
| Combined | 0.674 | 0.184 |
| Non-combined | 0.500 | **0.017** |
| Events | 0.596 | **0.034** |
| Objects | 0.782 | 0.270 |
| No-object | 0.461 | **0.006** |
| Manual indicative | 0.612 | 0.106 |
| Manual non-indicative | 0.648 | 0.127 |
| Left handed | 0.487 | 0.098 |
| Right-handed | 0.762 | 0.235 |
| Auditory long range | 0.754 | 0.239 |
| Auditory short range | 0.188 | **0.009** |
| Visual | 0.639 | 0.099 |
| Tactile | 0.197 | **0.003** |
| Multimodal with facial expression | 0.236 | **0.022** |
| Multimodal with high amplitude vocalization | 0.436 | **0.047** |
| Multimodal with low amplitude vocalization | 0.710 | 0.182 |
| Unimodal | 0.512 | **0.025** |
| Mutual attention present | 0.403 | **0.004** |
| Mutual attention absent | 0.320 | **0.007** |
| Piloerection | 0.547 | 0.131 |
| Penile erection | 1.686 | 0.095 |
| Close proximity | 0.221 | **0.001** |
| Far proximity | 0.600 | 0.081 |
| Heterogeneous | 0.802 | 0.320 |
| Homogenous | 0.350 | **0.001** |
| Non-repetitive | 0.584 | **0.040** |
| Repetitive | 0.504 | **0.015** |
| Single | 0.420 | **0.004** |
| Persistence | 0.586 | 0.145 |
| Rapid | 0.622 | 0.096 |
| Response present | 0.433 | **0.002** |
| Response absent | 0.298 | **0.001** |
| Response by visual or tactile gestural communication | 0.643 | 0.256 |
| Response by activity change | 0.480 | **0.004** |
| Response by vocalization | 0.462 | 0.078 |
| Dyadic repertoire size | 0.601 | **0.041** |
